# Cysteine string protein alpha accumulates with early pre-synaptic dysfunction in Alzheimer’s disease

**DOI:** 10.1093/braincomms/fcac192

**Published:** 2022-07-23

**Authors:** Huzefa Rupawala, Keshvi Shah, Caitlin Davies, Jamie Rose, Marti Colom-Cadena, Xianhui Peng, Lucy Granat, Manal Aljuhani, Keiko Mizuno, Claire Troakes, Beatriz Gomez Perez-Nievas, Alan Morgan, Po-Wah So, Tibor Hortobagyi, Tara L. Spires-Jones, Wendy Noble, Karl Peter Giese

**Affiliations:** 1 Department of Basic and Clinical Neuroscience, King’s College London, Institute of Psychiatry, Psychology and Neuroscience, 5 Cutcombe Road, London SE5 9RX, UK; 2 Centre for Discovery Brain Sciences and the UK Dementia Research Institute, The University of Edinburgh, 1 George Square, Edinburgh EH8 9JZ, UK; 3 Department of Neuroimaging, King’s College London, Institute of Psychiatry, Psychology and Neuroscience, 5 Cutcombe Road, London SE5 9RX, UK; 4 Department of Cellular and Molecular Physiology, Institute of Translational Medicine, University of Liverpool, Liverpool L69 3BX, UK; 5 Department of Neurology, ELKH-DE Cerebrovascular and Neurodegenerative Research Group, University of Debrecen, 4032 Debrecen, Hungary

**Keywords:** cysteine string protein alpha, pre-synapse, dystrophy, amyloid plaque, Alzheimer’s disease

## Abstract

In Alzheimer’s disease, synapse loss causes memory and cognitive impairment. However, the mechanisms underlying synaptic degeneration in Alzheimer’s disease are not well understood. In the hippocampus, alterations in the level of cysteine string protein alpha, a molecular co-chaperone at the pre-synaptic terminal, occur prior to reductions in synaptophysin, suggesting that it is a very sensitive marker of synapse degeneration in Alzheimer’s. Here, we identify putative extracellular accumulations of cysteine string alpha protein, which are proximal to beta-amyloid deposits in post-mortem human Alzheimer’s brain and in the brain of a transgenic mouse model of Alzheimer’s disease. Cysteine string protein alpha, at least some of which is phosphorylated at serine 10, accumulates near the core of beta-amyloid deposits and does not co-localize with hyperphosphorylated tau, dystrophic neurites or glial cells. Using super-resolution microscopy and array tomography, cysteine string protein alpha was found to accumulate to a greater extent than other pre-synaptic proteins and at a comparatively great distance from the plaque core. This indicates that cysteine string protein alpha is most sensitive to being released from pre-synapses at low concentrations of beta-amyloid oligomers. Cysteine string protein alpha accumulations were also evident in other neurodegenerative diseases, including some fronto-temporal lobar dementias and Lewy body diseases, but only in the presence of amyloid plaques. Our findings are consistent with suggestions that pre-synapses are affected early in Alzheimer’s disease, and they demonstrate that cysteine string protein alpha is a more sensitive marker for early pre-synaptic dysfunction than traditional synaptic markers. We suggest that cysteine string protein alpha should be used as a pathological marker for early synaptic disruption caused by beta-amyloid.

## Graphical abstract

**Graphic abstract:**
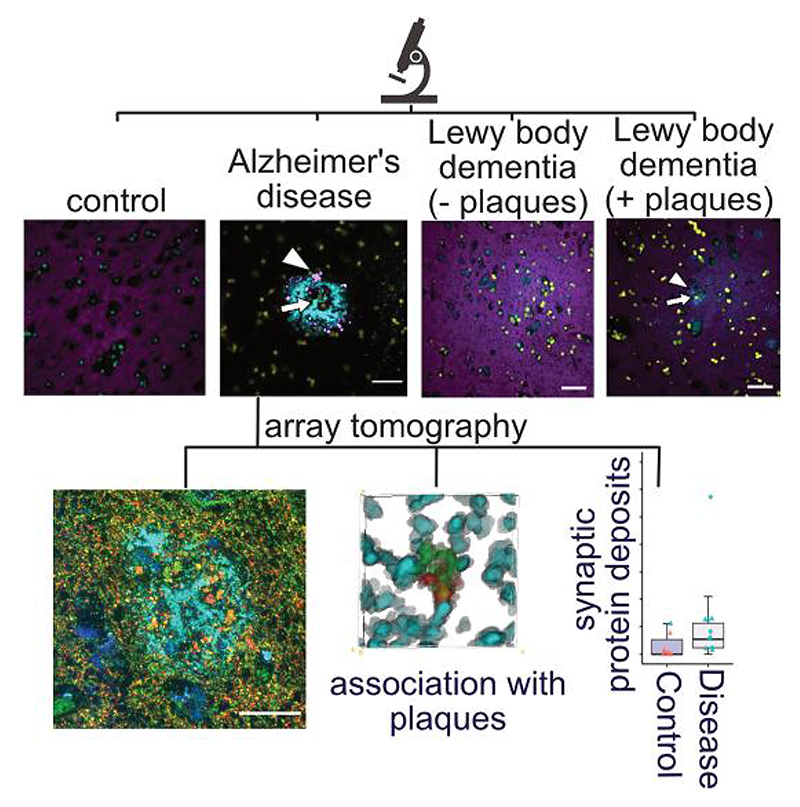

## Introduction

The pathological hallmarks of Alzheimer’s disease include extracellular beta-amyloid (Aβ) plaques and intracellular neurofibrillary tau tangles containing misfolded and aggregated tau, as well as dystrophic neurites, reactive glia, synapse loss, and brain atrophy predominantly in the forebrain.^1^ Among these pathological features, synapse loss correlates best with cognitive decline in Alzheimer’s disease.^2–7^ The mechanisms underlying synapse loss in Alzheimer’s disease are not currently known, but neuropathological analyses suggest that synapse loss precedes neuronal loss, with synapses lost from affected neurons prior to their degeneration.^8^ Expression of synaptophysin, a synaptic vesicle protein, is widely used to assess synaptic loss in post-mortem Alzheimer’s disease brain.^6^ However, in the hippocampus, alterations in the levels of another synaptic protein, cysteine string protein (CSP) alpha, occur prior to reductions in synaptophysin, suggesting that it represents an earlier and more sensitive marker of synapse degeneration in Alzheimer’s disease.^9^

CSPalpha is an evolutionarily conserved 27–34 kDa molecular co-chaperone protein localized at pre-synaptic vesicles in neurons.^10–12^ It is encoded by the *DNAJC5* gene and belongs to the CSP family, of which CSPalpha, beta and gamma are expressed in the brain.^11,13^ It contains a J domain, characteristic of the DnaJ/Hsp40 family of molecular chaperones that contains a HPD motif and allows binding to Hsc70/Hsp70 to facilitate protein refolding/disaggregation in the pre-synaptic compartment, and 11–14 cysteine residues (depending on species) that undergo palmitoylation, allowing CSPalpha to tether onto membranes and undergo ER-Golgi trafficking en route to synaptic vesicles.^14,15^ CSPalpha has multifunctional roles at the synapse that include maintaining proteostasis via stimulation of the ATPase cycle of Hsc70 and chaperone activity,^16–19^ regulating interactions with synaptic soluble, *N*-ethylmaleimide-sensitive attachment receptor proteins such as synaptosome-associated protein 25 (SNAP-25) for vesicular exocytosis^20^ and dynamin-1 for vesicular endocytosis and recycling.^21–23^ As such, CSPalpha has a significant role in the long-term maintenance of synaptic homoeostasis.^24^ Accordingly, a loss of CSPalpha in animal models leads to a degeneration and loss of synapses.^11,25–27^ More recently, CSPalpha was identified as an essential component of an unconventional misfolding-associated protein secretion (MAPS) pathway used to export neurodegenerative disease-associated misfolded proteins including tau, alpha-synuclein and TDP-43 either by an exosome-dependent release process or by an exosome-independent release process.^19,28,29^

Previous results from our laboratory have shown that CSPalpha immunoreactivity is reduced in the neurodegenerating regions of Alzheimer’s disease brain, such as the hippocampus and superior temporal gyrus, and is increased in the relatively spared cerebellar cortex.^9^ Furthermore, CSPalpha protein levels in synapses are reduced by over 40% in the temporal cortex of Alzheimer’s disease compared with control cases.^30^ These data led us to speculate that alterations in CSPalpha levels may be an early marker of synapse degeneration. To further explore this hypothesis, we examined the localization of CSPalpha in the BA9 prefrontal cortex region of a post-mortem Alzheimer’s disease brain. BA9 is one of the last areas to be affected in Alzheimer’s disease and displays enlarged synapses when synaptic degeneration first begins to occur.^2^ Here, using super-resolution microscopy and array tomography, we find for the first time that, together with widespread reductions in punctate synaptic CSPalpha labelling in these tissues, CSPalpha accumulates in proximity to both neuritic and diffuse Aβ plaques. These aberrant CSPalpha accumulations are largely distinct from hyperphosphorylated tau aggregates, axonal dystrophies and glial cells. CSPalpha was found to accumulate to a greater extent than other pre-synaptic proteins and at a comparatively great distance from the plaque core, indicating that CSPalpha is most sensitive to being released from presynapses at low concentrations of Aβ oligomers. These findings support an emerging body of literature suggesting that pre-synaptic degeneration is an early and key event in Alzheimer’s disease pathogenesis.^31^

## Materials and methods

### Post-mortem human tissue

Formalin-fixed, paraffin-embedded brain sections (7 μm thickness) were obtained from the London Neurodegenerative Diseases Brain Bank at King’s College London (REC approval 18/WA/0206). Tissues were obtained from Brodmann Area 9 (BA9) (dorsolateral and medial prefrontal cortices) from neurologically unimpaired individuals (Braak 0–II) (*n* = 11), moderate Alzheimer’s disease (Braak III–IV) (*n* = 5) and severe Alzheimer’s disease (Braak V–VI) (*n* = 10 sections), and hippocampus and cerebellum were secured from neurologically unimpaired (Braak 0–II) and subjects with severe Alzheimer’s disease (Braak VI) (*n*=3 for each group). Temporal cortex sections were also obtained from neurologically unimpaired donors (Braak 0–II) and those with fronto-temporal lobe dementia (FTLD) and dementia with Lewy bodies (DLB) (*n*=5 for each group). Resin-embedded fresh tissue sections (70 nm thickness) for array tomography were obtained from the MRC Edinburgh Brain Bank and from neurologically unimpaired donors (Braak 0–II) (*n*=8) and those with severe Alzheimer’s disease (Braak V–VI) (*n*=10) (Supplementary Table 1). The use of post-mortem human tissue for array tomography has been reviewed and approved by the Edinburgh Brain Bank Ethics Committee and the Academic and Clinical Central Office for Research and Development Medical Research Ethics Committee, a joint office of the University of Edinburgh and NHS Lothian (approval 15-HV-016). The Edinburgh Brain Bank is a Medical Research Council-funded facility with research ethics committee approval (11/ES/0022).

Cases were sex- and age-matched. Tissue was assessed for post-mortem delay and quality by examining protein degradation to ensure that these parameters are comparable between groups, as in previous studies.^9,32^ All human tissue was handled according to Human Tissue Authority and local regulations.

### Preparation of synaptoneurosomes and western blotting

Total, cytosolic and synaptoneurosome fractions were isolated from the post-mortem temporal cortex as described previously.^7^ Briefly, synaptoneurosomes were prepared from ~250 mg of frozen tissue (grey matter). Tissue was homogenized in 1.5 ml of ice-cold Buffer A (25 mM HEPES, pH 7.9, 120 mM sodium chloride, 5 mM potassium chloride, 1 mM magnesium chloride, 2 mM calcium chloride, 1 mM dithiothreitol, protease inhibitors and phosphatase inhibitors) using a Teflon-glass mechanical tissue grinder at 170 rpm and filtered through 80 μm pore filters. A portion of the filtrate was collected, supplemented with 1.5% sodium dodecyl sulphate (SDS), boiled for 5 min, and centrifuged at 15 000 g for 15 min, to give the total protein fraction. The remaining sample was filtered through 5 μm pore filters and centrifuged at 1000 g for 10 min to pellet synaptoneurosomes. The supernatant was collected as a cytosolic extract, which was further centrifuged at 100 000 g for 30 min to remove microsomes. The synaptoneurosome pellet was washed once with cold Buffer A and centrifuged again at 1000 g for 10 min. The pellet was extracted with Buffer B (50 mM Tris pH 7.5, 1.5% SDS, 2 mM dithiothreitol) (0.5 ml) and boiled for 5 min. After centrifugation at 15 000 g for 15 min, the supernatant was collected as the synaptoneurosome fraction. Protein concentrations of samples were determined using a bicinchoninic acid assay protein assay kit (Thermo Fisher Scientific) and Ponceau Red staining of membranes. Equal protein amounts of total, synaptic and cytoplasmic fractions were electophoresed on 10% Tris-glycine SDS-polyacrylamide gels, (Invitrogen), transferred to a 0.45 μm nitrocellulose membrane (Millipore, Burlington, MA, USA) and immunoblotted using standard methods. The primary antibodies were CSPalpha (AB1576, Merck Millipore) and synaptophysin (sc17750; Santa Cruz). Bound antibodies were imaged using an Odyssey CLX instrument (LI-COR Biosciences) and band intensities were quantified using the Image Studio Software (LI-COR Biosciences). The amount of CSPalpha in the total and synaptoneurosome fractions was normalized to the amount of synaptophysin in the same sample.

### Immunohistochemistry

Immunohistochemistry with 3′ 3-diaminobenzidine staining was performed as previously described,^9^ using a primary antibody against CSPalpha (1:500 AB1576, Merk Millipore). Brightfield images were captured using an Olympus Slide scanner (VS120) and viewed using the Olympus Viewer software (VS120).

For immunofluorescence, rehydrated sections were washed in Tris-buffered saline (TBS) containing 0.01% Tween20 (TBS-T) and non-specific binding reduced by blocking for 1 h in 10% normal goat serum (NGS) (Sigma) in TBS before incubating with primary antibodies (Supplementary Table 2) at 37° C for 1 h. Following washes in TBS-T, fluorescently tagged secondary antibodies (Supplementary Table 3) were applied for 45 min at room temperature. After further washing in TBS-T, sections were exposed to Sudan black (Sigma, UK) for 10 min to reduce autofluorescence. Sections were washed and coverslips mounted using a Vectashield mounting medium containing 4′,6-diamidino-2-phenylindole (DAPI) (H-1200, Vectashield). Images were either viewed or captured by using a Leica DM5000B fluorescence microscope and CTR5000 camera (Leica Microsystems, Germany) or by using a Nikon Ti-E Live Cell Three Camera microscope, using the 20× and 40× objective lenses. For confocal imaging, a Nikon Eclipse Ti inverted spinning disc confocal microscope (Nikon Instruments, UK) with a Yokogawa CSU-1 disc head and Andor iXon EMCCD camera and either a 20×, 40× or 60× oil objective lens (Nikon Instruments, UK) were used. Parameters including laser settings, camera setup and calibration were kept constant during image capture. Image ‘Z’ stacks were acquired covering a total ‘Z’ depth of 12 μm. Maximum intensity and volume projection were produced from collapsing the Z stacks in the NIS-Elements AR software (Nikon Instruments, UK). The 3D equatorial diameter (diameter of the sphere with the same volume as the measured object) was measured using the following equation (Nikon Elements, Nikon), from which the volume was determined as a minimum threshold of 8 μm^3^. Images were stored as TIFF files.

$${\text{Equatorial}\;\text{diameter}\;}_{3\text{D}}={\left(\frac{6\times \;\text{Volume}\;}{\pi }\right)}^{1/3}$$

For super-resolution imaging, an instant Structured Illumination Microscope (iSIM) was used with a Vt-iSIM scan head equipped with the Hamamatsu Flash4.0 sCMOS camera and by using an 100X/1.49NA oil objective. Image ‘Z’ stacks were acquired covering a total ‘Z’ depth of 12 μm. Volume projection images were produced in the NIS-Elements AR software (Nikon Instruments, UK).

### Array tomography

Fresh post-mortem human brain tissue from BA9 was embedded as previously described in LR white resin.^33^ Resin-embedded tissue blocks were cut into ribbons of 70 nm serial sections using an ultracut microtome (Leica) with a Histo Jumbo Diamond Knife (Diatome, Hatfield, PA, USA) and collected onto gelatine-coated coverslips. A hydrophobic barrier was drawn around each ribbon. The ribbons were rehydrated in 50 mM glycine for 5 min and exposed to antigen retrieval [1 mM ethylenediaminetetraacetic acid (pH: 8.0)/0.05% Tween-20 treatment using a pressure cooker on steam setting] for 1 min. Array ribbons were washed in TBS and blocked for 30 min with 0.1% fish skin gelatine and 0.05% Tween-20, before incubation with primary antibodies (Supplementary Table 2) overnight at 4°C. The ribbons were washed in TBS and the appropriate Alexa Fluor-conjugated secondary antibodies (Supplementary Table 3) were applied for 30 min. Following further washing in TBS, sections were counterstained with 0.01 mg/ml DAPI, washed in TBS and mounted onto coverslips using immunomount (DAKO). A ‘no primary’ negative control was included with each experiment.

Images were acquired with a Zeiss Axio Imager Z2 upright epi-fluorescence microscope equipped with a CoolSnap digital camera and by using a high-resolution 63× oil 1.4NA Plan Apochromat objective and imaged employing the AxioImager software with array tomography macros (Carl Zeiss, Ltd, Cambridge, UK). The images were manually acquired from the area of interest on each serial section of the ribbon using either Aβ plaques or DAPI-stained nuclei as a reference marker. Image stacks were created and aligned for regions of interest in the neuropil using image J and the MultiStackReg custom plugin.^34^ Binarized image stacks were combined using different thresholding algorithms in Image J. For synaptic image stacks, this allowed the detection of both high- and low-intensity synapses in an automated fashion. Using custom MATLAB scripts, the images were manually thresholded to detect synaptic puncta and CSPalpha accumulations present around Aβ plaques. Synaptic density was calculated using the thresholded images to remove background noise (only objects present in two or more stacks were retained) and to calculate the co-localization of Aβ with CSPalpha and synaptophysin (pre-synaptic terminals). The relative co-localization was calculated as a 10% minimum overlap of the number of co-localization puncta or deposits/sum total puncta for each protein of interest. In addition, MATLAB scripts were used to measure the density of pre-synaptic accumulations (both CSPalpha and synaptophysin) from the centre of the Aβ plaque core (plaque distance) using bin sizes of 0–10, 10– 20, 20–30 and 30–40 μm.

Custom array tomography analysis macros are freely available on GitHub https://github.com/arraytomographyusers/Array_tomography_analysis_tool.

### Animals

Genotyped, heterozygous 5xFAD mice (*n*=3) and WT littermates (*n*=4) were obtained from a colony of breeding mice, established at the Institute of Psychology, Psychiatry and Neuroscience. 5xFAD mice generated on a C57/B6 x SJL background strain express Alzheimer’s disease-linked mutant human *APP* [Swedish (K670N/M671L), Florida (I716V) and London (V717I)] and *PSEN1* (M146L and L286V) transgenes.^35^ Mice were group-housed under standard laboratory conditions with food and water *ad libitum*. All animal work was conducted in accordance with the UK Animals (Scientific Procedures) Act 1986 and the European Directive 2010/63/EU under UK Home Office Personal and Project Licenses and with agreement from the King’s College London (Denmark Hill) Animal Welfare and Ethical Review Board.

### Mouse tissue processing and immunohistochemistry

WT and 5xFAD mice were anaesthetized with an intraperitoneal injection of Euthatal® (Merial, Toulouse, France) and intra-cardially perfused with phosphate-buffered saline (PBS) followed by 4% paraformaldehyde in PBS. The brains were post-fixed in 4% PFA overnight and cryoprotected for 24 h in 30% sucrose in PBS. The brains were snap-frozen in isopentane and stored at −80 °C until they were ready for cutting and processing. Tissue was mounted onto stages using a cryo-embedding compound, and 30 μm coronal sections were cut.

A one in four series of sections from WT and 5×FAD mice was immunohistochemically labelled as previously described,^36^ using primary antibodies against CSPalpha (1/2000, AB1576, Merck Millipore), 6E10 (1/2000, 803001, Biolegend), synaptophysin (1/1000, sc-17750, Santa Cruz) and synaptosomal-associated protein, 25 kDa (SNAP-25, 1/1000, sc-20038, Santa Cruz). After washing with PBS, sections were incubated in PBS containing 0.1% Triton X-100 with appropriate secondary antibodies for 2 h at room temperature. After washing with PBS, the sections were mounted on poly-l-lysine slides (VWR, DE), air-dried and coverslips were added using a Vectashield mounting medium containing DAPI (H-1200, Vectashield). Some additional sections were stained with Thioflavin-S treatment by placing them into a Thioflavin-S solution for 8 min. The sections were rinsed in 50% ethanol and TBS before air drying and mounting.

Fluorescence images were captured using an Olympus whole slide scanner VS120 with a 2/3”CCD camera, motorized XY stage with automatic control. An image overview of the sections was taken using a 2×objective lens. High-resolution images at 60×magnification were captured using spinning disc confocal microscopy as previously for human tissues.

### Statistical analysis

Array tomography image stacks were analysed using MATLAB (version R2019a, Mathworks Inc., USA) and Image J. If required, data were transformed using either the Tukey transformation or Box–Cox transformation (log-likelihood or Guerro methods), and Shapiro–Wilk’s test was used to test for normality. Data were statistically analysed using linear mixed-effects modelling with Type I−III one-way ANOVA with Satterthwaite’s degrees of freedom method. Outliers were included in the data set. Data in bar graphs were presented as either mean ± standard error of the mean (SEM) or median with box plots for interquartile ranges. All statistical analyses were performed in R (http://www.r-project.org).

Data from western blots were analysed using ANOVA, followed by Tukey’s multiple comparison test or two-way ANOVA with repeated measures, and *post hoc* Sidak multiple comparisons test for parametric data. Data were expressed as mean ±SEM unless otherwise stated. Statistical analysis was conducted using the Prism 8 (Graphpad) software.

## Results

### CSPalpha forms amorphous deposits in Alzheimer’s disease brain

**Figure 1:**
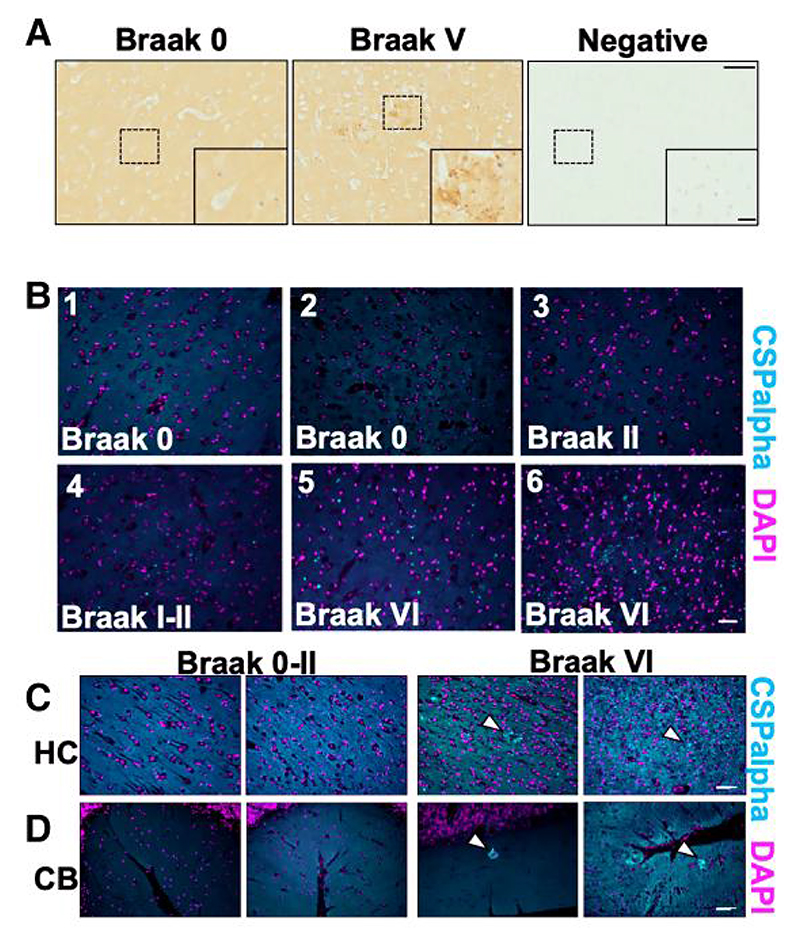
Focal spots and amorphous CSPalpha-positive deposits identified in Alzheimer’s disease brain. Representative images from fixed post-mortem human BA9 sections from age-matched (**A**) Braak 0–II (*n*=6) and severe Alzheimer’s disease (Braak V–VI) (*n*=5) cases probed with an anti-CSPalpha antibody. The higher magnification represents the area indicated by the dashed box. The detection of CSPalpha expression (brown) in Alzheimer’s disease brain occurred in dense focal spots and larger amorphous deposits. Nuclei were counterstained with haematoxylin (blue). A negative control where no primary antibody was used confirmed the specificity of labelling. Scale bars are 100 μm (low magnification image) and 20 μm (high magnification inset). (**B**) Representative epi-fluorescent images of 7 μm thick post-mortem human BA9 brain sections from Braak stages 0−VI immunolabelled using an antibody against CSPalpha (cyan) with nuclei stained with DAPI (magenta). (*n*=5 cases per group.) Scale bar 50 μm. CSPalpha accumulations were apparent within the grey matter. (**C**) Representative images of post-mortem human hippocampus (HC) and (**D**) cerebellum (CB) from two Alzheimer’s disease (Braak Stage VI) and two control (Braak Stages 0–II) brain sections immunolabelled using an antibody against CSPalpha (cyan). Dotted lines in (**C**) indicate focal spots and amorphous deposits of CSPalpha. DAPI was used to stain nuclei (magenta). The white arrow heads indicate CSPalpha accumulations. Scale bar 50 μm. (*n*=3).

Formalin-fixed, paraffin-embedded sections from five severe Alzheimer’s disease (Braak V–VI) and six control (Braak 0–II) brains were immunolabelled with an antibody against CSPalpha. In Alzheimer’s disease, we noted striking amorphous accumulations of CSPalpha, particularly within the grey matter in BA9, which were largely absent in control tissues. Some control and Alzheimer’s disease tissues also showed smaller bright focal spots of CSPalpha (Fig. 1A and B). Since the focal spots appeared to increase in number as disease worsened, we believed that they might be precursors to more mature amorphous deposits. Bright focal spots and amorphous deposits of CSPalpha were also identified in the hippocampus, a region primarily and severely affected in Alzheimer’s disease.^37^ The cerebellum, which has a lower plaque burden than the cortex and hippocampus in Alzheimer’s disease,^38^ also showed amorphous accumulations and bright focal spots of CSPalpha (Fig. 1C and D).

We also probed the western blots of lysates and synapto-neurosome fractions from Braak Stage 0–II, III–IV or V–VI cases with antibodies against CSPalpha and synaptophysin. CSPalpha was detected as a single main band of ~34 kDa. CSPalpha levels were normalized to synaptophysin levels in the same sample to control for synapse loss. We found no change in CSPalpha levels relative to synaptophysin in Braak Stage III–IV or V–VI total homogenates relative to controls (Braak Stage 0–II). The synaptoneurosome fraction showed an apparent reduction in CSPalpha levels relative to synaptophysin, but this did not reach statistical significance (*P =* 0.06;Supplementary Fig. 1).

The N-terminal domain of CSPalpha includes a serine at amino acid position 10 that is phosphorylated by protein kinase A and/or protein kinase B^39,40^(Supplementary Fig. 2A). Increased phosphorylation of CSPalpha at Ser10 was identified in a phosphoproteomics data set of cortical synaptoneurosomes from APP/PS1 over-expressing mice.^41^ Phosphorylation at this site causes a structural destabilization of CSPalpha at its N-terminus that affects CSPalpha functions in synaptic vesicle fusion and releases kinetics.^39,42^ In particular, it was shown that the phosphorylation of CSPalpha at Serine (Ser10) is required for the extracellular release of tau.^21^ As CSPalpha itself is released alongside the client proteins in this MAPS process, one would, therefore, predict that extracellular CSPalpha in the brain would be preferentially phosphorylated at Ser10. Since the accumulations of CSPalpha that we observed in Alzheimer’s disease brain might reflect extracellular CSPalpha, we used a specific CSPalpha pSer10 antiserum^18,19^(Supplementary Fig. 2B and C). We observed multiple CSPalpha bands in wild-type mouse brain lysates and in samples from human hippocampus spanning from ~29–34 kDa. In addition to phosphorylation, CSPalpha is subject to palmitoylation. Others have shown that CSPalpha is apparent as a ‘stepladder’ on immunoblots with bands ranging from 27 to 34 kDa that can be collapsed to the basal 27 kDa molecular weight upon deacylation of protein thiols.^13^ The banding pattern that we observed with mouse and human lysates likely reflected a differential modification of CSPalpha in these samples. Using the CSPalpha pSer10 antiserum, we found that at least some of the CSPalpha deposited in Alzheimer’s disease brain was phosphorylated (Supplementary Fig. 2D).

### CSPalpha deposits are associated with Aβ pathology

**Figure 2:**
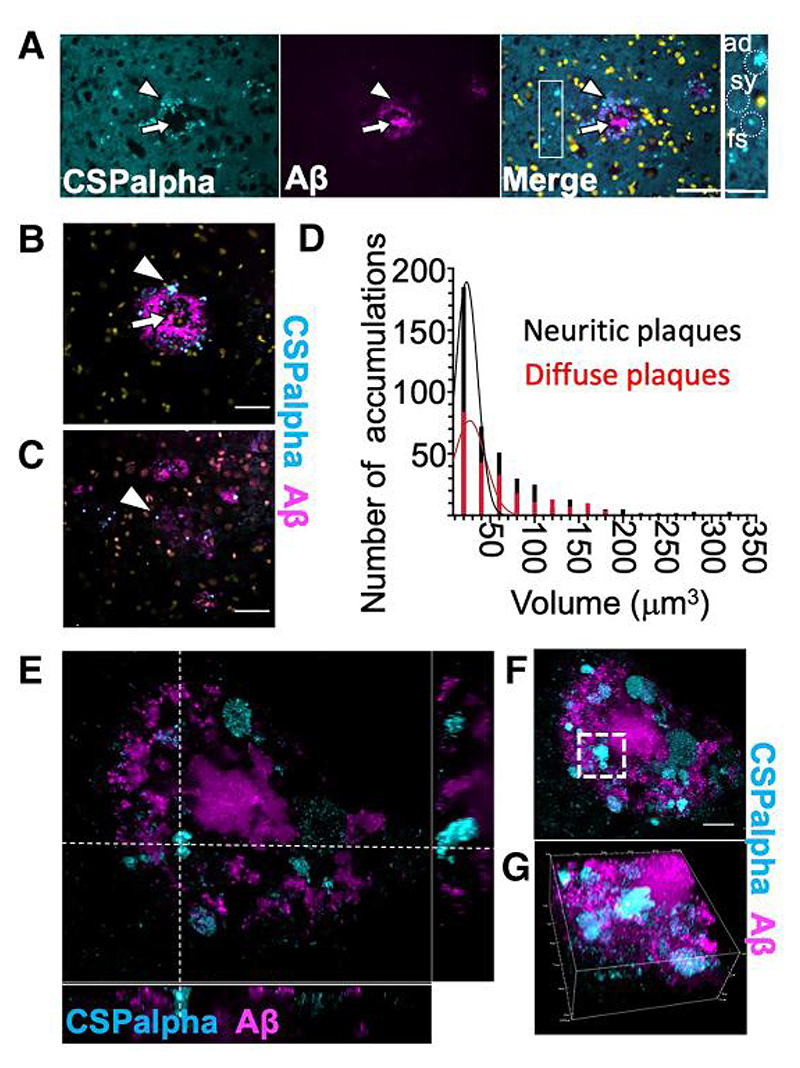
CSPalpha-positive accumulations in Alzheimer’s disease brain. (**A**) Representative maximum intensity projection images of sections from a Braak VI post-mortem human Alzheimer’s disease brain (BA9), co-labelled with antibodies against CSPalpha (cyan) and Aβ (6E10) (magenta). Merge of images shown together with DAPI staining (yellow). The arrow heads indicate amorphous deposits of CSPalpha, and the complete arrows indicate the plaque core. Scale bar 50 μm. (*n*=3 Braak VI brains.) The inset shows relative scales of synaptic CSPalpha (sy), several small focal spots (fs), one larger fs and one amorphous deposit (ad), examples indicated by dotted circles. Scale bar 25 μm. Representative spinning disc confocal images (projection of a stack of 10 optical sections) show amorphous CSPalpha deposits (cyan, arrowhead) in Braak VI post-mortem human Alzheimer’s disease brain (BA9) associated with (**B**) Aβ-positive neuritic and (**C**) diffuse plaques (magenta). Nuclei are stained with DAPI (yellow). Synaptic neuropil staining (background) was subtracted from measurements. Scale bar 25 μm. The complete arrows indicate the plaque core. (**D**) Histogram showing quantification of the number of CSPalpha accumulations and their volumetric measurements for both neuritic cored (*n*=101) and diffuse plaques (*n*=54). (*n*=5 brains). (**E**) Representative super-resolution 25 × 26 μm iSIM image section showing orthogonal views of amorphous CSPalpha deposits and focal spots in association with a large neuritic cored Aβ plaque. The white segmented line intersects an amorphous CSPalpha deposit. (**F**) Maximum intensity projection of 58 optical sections of the same orthogonal field of view. The white segmented box shows the same CSPalpha amorphous deposit. Scale bar 10 μm. (**G**) Volumetric 3D maximum intensity projection of an isolated CSPalpha deposit. Magnification 100×. Volume of box: 25 μm×26 μm×II μm.

The pattern of amorphous CSPalpha deposits suggested that they might form at the periphery of Aβ plaques. To confirm this, sections were co-labelled with antibodies against CSPalpha and Aβ (6E10). These data showed that amorphous CSPalpha deposits and the bright focal spots were common in the periphery of neuritic plaques, without infiltrating the plaque core (Fig. 2A). High-resolution spinning disc confocal imaging confirmed that although no amorphous deposits of CSPalpha were found in areas distant from Aβ plaques, some focal spots were apparent in non-plaque areas (Fig. 2A). Both types of CSPalpha accumulations were associated with diffuse (low staining intensity and diffuse spread of Aβ deposition) and neuritic cored plaques (consisting of a higher staining intensity and a neuritic core)^43^ (Fig. 2B and C), and bright focal spots appeared more prominent near diffuse plaques (Fig. 2C). Quantitative volumetric measurement of CSPalpha accumulations was used to determine that the volume of the majority of CSPalpha structures ranged between 10 and 50 μm^3^ (56.4%, mean size 24.8 ±0.57 μm^3^), which correspond to the bright focal spots of CSPalpha, with fewer larger-sized amorphous deposits (50–100 μm^3^–22.6%, mean size 71.2±1.16 μm^3^ and >100μm^3^–14.8%, and mean size 164±5.94 μm^3^). The volume of the CSPalpha accumulations varied across neuritic (*n* = 440 objects, *n* = 101 plaques, maximum object volume 328μm^3^, and median object volume 30.9 μm^3^) and diffuse plaques (*n* = 241 objects, *n* = 54 plaques, maximum object volume 346μm^3^, and median object volume 39.4μm^3^) (Fig. 2D). However, no differences were observed between the type of plaque and the mean size of CSPalpha accumulations. Instant Structured Illumination Microscopy (iSIM) showed that amorphous CSPalpha deposits and focal spots were clearly visualized at the periphery of larger Aβ deposits (Fig. 2E–G).

**Figure 3:**
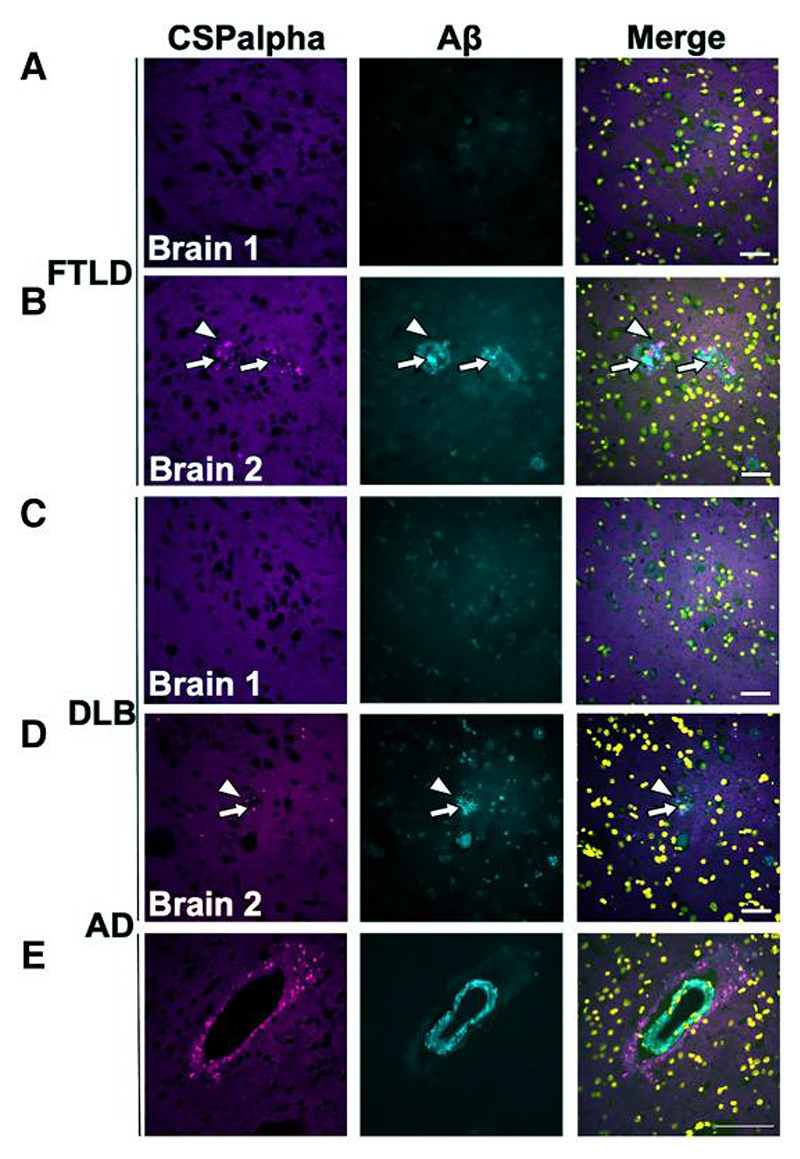
CSPalpha accumulations in association with Aβ deposits. Representative immunofluorescence images of post-mortem temporal cortex brain sections from cases with (**A**) FTLD without Aβ plaques, (**B**) FTLD with Aβ plaques, (**C**) DLB without Aβ plaques and (**D**) DLB with Aβ plaques. Sections were immunolabelled with antibodies against CSPalpha (magenta), Aβ (6E10) (cyan) and DAPI (yellow) (*n*=5 per group). The arrow heads indicate amorphous CSPalpha deposits in (**B**) and focal spots in (**D**). The complete arrows indicate the plaque core. Scale bar 50 μm. (**E**) Representative temporal cortex section from Braak stage VI post-mortem Alzheimer’s disease brain, showing CSPalpha focal spots in proximity to an artery with a vessel wall disrupted by Aβ deposits and dysphoric angiopathy. Brain sections were co-labelled with antibodies against CSPalpha (magenta) and Aβ (6E10) (cyan). DAPI (yellow) was used to stain nuclei. Scale bar 100 μm.

Next, we wanted to explore if CSPalpha deposits are specific to Aβ pathology in Alzheimer’s disease or are also observed in related neurodegenerative diseases. Sections of the temporal cortex from FTLD and DLB brains were co-labelled with antibodies against CSPalpha and Aβ (6E10). Interestingly, three FTLD and DLB brains were found to contain amyloid plaque-associated CSPalpha accumulations in line with their mixed-Alzheimer’s disease pathological diagnosis, the density of which was substantially lower than what was observed in Alzheimer’s disease (Fig. 3B and D). The FTLD brains showed both amorphous CSPalpha deposits and bright focal spots (Fig. 3B), whereas in DLB, only bright focal spots were observed (Fig. 3D). On the other hand, two FTLD and DLB brains that did not show Aβ deposition also did not display CSPalpha accumulations (Fig. 3A and C). This shows that CSPalpha accumulations are determined by the presence of Aβ deposits, independent of disease type. Interestingly, the focal spots of CSPalpha were also found to associate with cerebrovascular Aβ deposits in some cortical arteries and arterioles in Alzheimer’s disease, with evidence of dysphoric angiopathy (flame-like Aβ deposits that radiate into the neuropil from the vessel wall) (Fig. 3E).

### Plaque-associated CSPalpha accumulations in 5xFAD transgenic mouse brains

**Figure 4:**
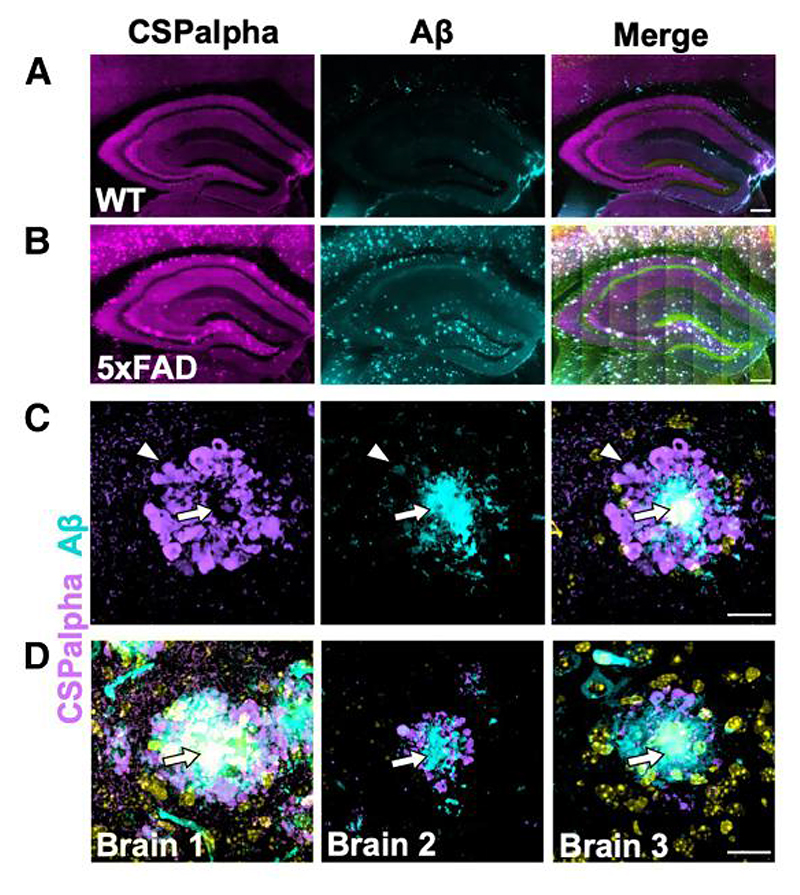
Aβ plaque-associated CSPalpha accumulation in 5×FAD transgenic mice. Representative low magnification images of the hippocampus from a minimum of three consecutive sections from female (**A**) wild-type (WT) mouse (*n*=4) and (**B**) 5×FAD transgenic mouse (*n*=3) brains (~6 months old) co-labelled with CSPalpha (magenta) and Aβ (cyan) antibodies. DAPI (yellow) was used to stain nuclei. Scale bar 200 μm. (**C**) Representative higher magnification image of CSPalpha immunoreactivity in proximity to Aβ plaques in 5xFAD brain. The arrow heads indicate amorphous CSPalpha deposits, and the complete arrows indicate the plaque core. Scale bar 20 μm. (**D**) Representative higher magnification images of hippocampal sections taken from three female 5xFAD mice (~6-month-old). The merged images show CSPalpha immunoreactivity (magenta) in proximity to individual 6E10-positive Aβ plaques (cyan). DAPI stain (yellow) was used for nuclei. Scale bar 20 μm.

To buttress the fact that CSPalpha accumulations are associated with Aβ plaques, CSPalpha distribution was examined in 5xFAD mice. These mice harbour FAD-causing mutations in human *APP* and *PSEN1* genes, progressively develop Aβ plaques from as early as 3 months of age and show age-dependent neuroinflammation, synaptic abnormalities, neuronal loss and cognitive decline.^35^ Six-month-old 5xFAD mice exhibited prominent Aβ labelling marking intraneuronal APP and Aβ that was absent in the hippocampus of WT mice (Fig. 4A and B). Increased labelling of CSPalpha was found within the dentate gyrus hilus, intrapyramidal bundle, stratum lucidum and subiculum of the hippocampus (Fig. 4A and B) from as early as 3 months in the 5xFAD brain. These data suggest that CSPalpha accumulations form during the early plaque deposition, prior to substantial synapse loss in 5×FAD mice^35^ (Supplementary Fig. 3). The subiculum region showed the highest density of Aβ deposits at 3 months of age, where virtually all 6E10-positive Aβ plaques were decorated with amorphous CSPalpha deposits and focal spots. At 6 months of age, amorphous deposits and focal spots of CSPalpha were found in association with Aβ deposits, mirroring the CSPalpha deposition found in human Alzheimer’s disease brain (Fig. 4C and D). Some small Aβ-positive structured showed no accompanying CSPalpha deposits, but rather were associated with bright CSPalpha spots. Similarly, some bright focal spots of CSPalpha were found in areas distal from Aβ plaques. These data suggest that substantial Aβ deposits are required for the accumulation of large amorphous deposits of CSPalpha in neurodegenerative disease.

### CSPalpha deposits do not co-localize with plaque-associated tau, glia or axonal dystrophies

**Figure 5:**
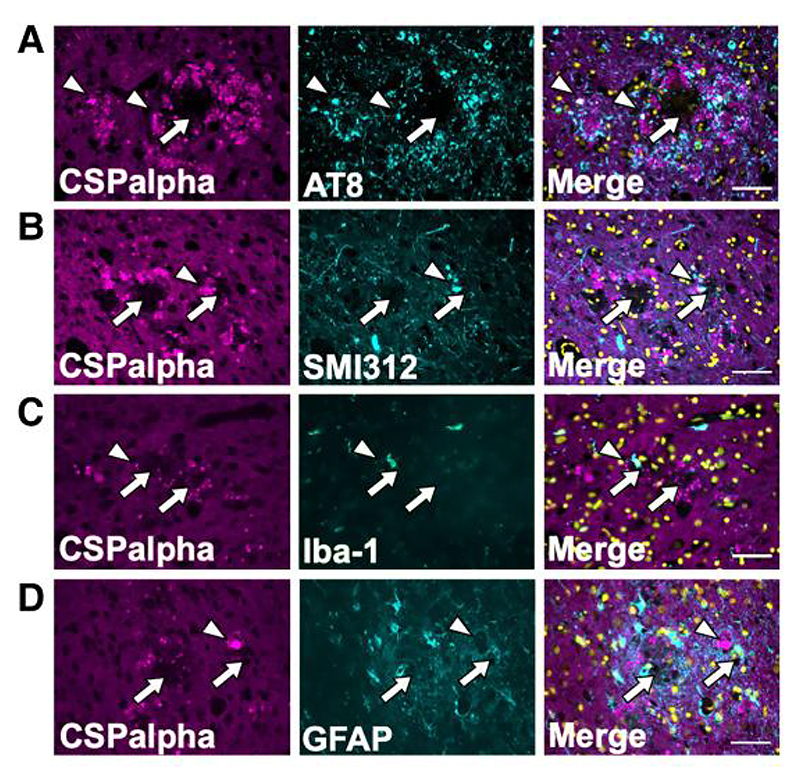
Amorphous CSPalpha deposits do not co-localize with plaque-associated tau, glia or axonal dystrophies. Representative maximum intensity projection images of sections from a Braak VI post-mortem human Alzheimer’s disease brain (BA9), with two consecutive sections co-labelled with antibodies against CSPalpha (magenta) and (**A**) AT8 (tau phosphorylated at Serine202/Threonine205) (cyan), (**B**) SMI312 (neurofilament/dystrophic neurites) (cyan), (**C**) Iba-I (microglia) (cyan) and (**D**) GFAP (activated astrocytes) (cyan). Merged images shown together with DAPI staining (yellow). CSPalpha immunoreactivity is localized in proximity to Aβ deposits and overlaps with some but not all dystrophic neurites, AT8-positive tau and glial cells (e.g. white arrowheads). The complete arrows indicate the plaque core. Scale bar 50 μm. (*n*=3 Braak VI brains).

Next, it was important to determine if CSPalpha accumulates together with other plaque-associated features. CSPalpha has been implicated in the release and spread of tau,^19,29^ and therefore, consecutive sections of BA9 from Braak VI brain were immunolabelled for CSPalpha and AT8 (tau phosphorylated at Ser199/Ser202/Thr205). AT8-positive labelling was observed as neuropil threads, pre-tangles, NFTs and neuritic dystrophies; however, these were only rarely co-localized with amorphous CSpalpha deposits (Fig. 5A). Axonal dystrophies are typical in the vicinity of amyloid plaques in Alzheimer’s disease.^44,45^ We speculated that these might be the site of CSPalpha accumulations and so we performed co-immunofluorescence of CSPalpha with the anti-neurofilament marker, SMI312. However, again we found that CSPalpha rarely overlapped with SMI312-positive immunoreactive inter-axonal enlargements within the same area (Fig. 5B). This indicates that few CSPalpha accumulations localize within degenerating axons that encircle Aβ deposits in Alzheimer’s disease brain.

Reactive astrocytes and microglia surround Aβ deposits^46^ and phagocytose protein aggregates and degenerating synapses.^47,48^ To determine if CSPalpha accumulates following the internalization of degenerating pre-synapses by glia, sections were co-labelled with antibodies against CSPalpha and either glial fibrillary acidic protein (GFAP), a marker of reactive astrocytes, or ionized calcium binding adapter molecule 1 (Iba-1), a microglial marker. Numerous GFAP-positive astrocytes and Iba-1-positive microglia were identified surrounding the plaques. However, there was no apparent co-localization of these glial markers with CSPalpha (Fig. 5C and D).

### CSPalpha accumulations localize with synaptic markers in proximity to Aβ plaques

**Figure 6:**
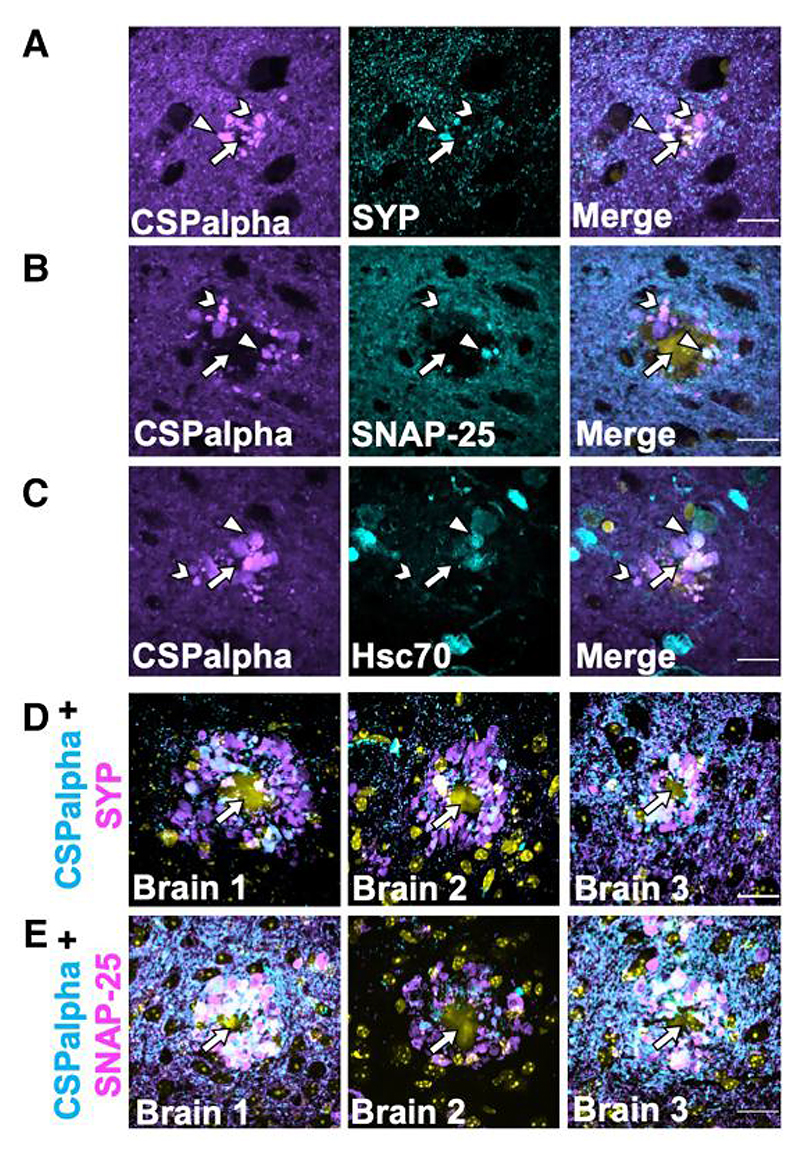
Few CSPalpha accumulations co-localize with pre-synaptic terminal structures in proximity to amyloid plaques. Representative images of sections from BA9 of Braak Stage VI human Alzheimer’s disease brains co-labelled with antibodies against (**A**) CSPalpha (magenta) and synaptophysin (cyan), (**B**) SNAP-25 (cyan), or (**C**) Hsc-70 (cyan). DAPI (yellow) was used to stain nuclei. CSPalpha immunoreactivity overlaps with some synaptophysin-positive pre-synaptic structures, likely dystrophic terminals, as well as SNAP-25, and Hsc-70. The arrow heads indicate co-localizing CSPalpha accumulations, the chevrons indicate isolated CSPalpha accumulation and the complete arrows indicate the plaque core. Scale bar 20 μm. (*n*=3 Braak VI brains, minimum of three sections per labelling), (**D**) Representative images of hippocampal sections taken from three 5xFAD mice (~6-month-old). The images show some but not all CSPalpha accumulations (magenta) co-labelling with pre-synaptic markers synaptophysin (SYP) (cyan) and (**E**) SNAP-25 (cyan) in 5xFAD mice. DAPI staining (yellow) was used to identify nuclei. Yellow autofluorescence also detected the Aβ plaque core. The complete arrows indicate the plaque core. Scale bar 20 μm.

Previous studies have reported the disruption and disorganization of synapses in Alzheimer’s disease, whereby pre-synaptic proteins have been found to accumulate within degenerating and dystrophic pre-synaptic terminals, termed ‘pre-synaptic dystrophies’, which are incorporated at the earliest stages of Aβ plaque formation.^4,49–51^ These pre-synaptic structures appear similar in shape and size to the plaque-associated CSPalpha deposits found here. As such, it was important to explore the possibility that CSPalpha accumulates in association with other synaptic markers. Synaptophysin, a pre-synaptic protein, is present on CSPalpha-bound synaptic vesicles,^52^ and so it is possible that these proteins co-localize within the pre-synaptic terminal. CSPalpha and synaptophysin were immunolabelled in Braak VI BA9 post-mortem brain sections and there was some co-localization of these proteins. Amorphous deposits of both CSPalpha and synaptophysin were evident in the vicinity of neuritic cored plaques and as described in previous reports for synaptophysin.^51,53–56^ Focal puncta of CSPalpha showed a lesser extent of co-localization with synaptophysin (Fig. 6A). Similar synaptophysin deposits were apparent surrounding Thioflavin-S-labelled plaques in 5×FAD brain (Supplementary Fig. 4). In human brain, CSPalpha accumulations appeared more abundant than those observed for synaptophysin.

SNAP-25 is an interacting partner of CSPalpha at presynapses that has previously been shown to accumulate in Alzheimer’s disease brain.^54,57^ SNAP-25 labelled the pre-synaptic neuropil as previously reported,^58^ and some, but not all, SNAP-25 immunoreactive structures also labelled with CSPalpha, particularly for the larger amorphous deposits (Fig. 6B). Similarly, heat shock cognate 71 kDa protein (Hsc70), a cytosolic chaperone protein known to interact with CSPalpha^22^ and mediate tau release,^29^ showed some co-labelling with CSPalpha, particularly within the proximity of presumed plaque cores (Fig. 6C). Similar findings were obtained when hippocampal sections from 5×FAD mice were co-labelled with antibodies against CSPalpha, synaptophysin and SNAP-25 (Fig. 6D and E) in the proximity of Aβ plaques.

To better visualize the association of CSPalpha accumulations with other synaptic markers in Alzheimer’s disease, array tomography (AT)59 was used, as previously reported for human post-mortem tissue.^33,60^ AT overcomes the difficulties of axial, or z-resolution, of traditional imaging techniques. Using custom MATLAB scripts, images were manually thresholded to detect synaptic puncta and CSPalpha accumulations present around the Aβ plaques. Synaptic density was calculated using the thresholded images to remove background noise (only objects present in two or more stacks were retained) and to calculate the co-localization of Aβ with CSPalpha and synaptophysin (pre-synaptic terminals). This allowed synapses labelled at both high and low intensity to be measured in a semi-automated and unbiased manner. Plaque cores were segmented with a fixed threshold to find the edge of dense plaques at a distance from plaque measurements. Objects that were not present in more than one consecutive section were removed to reduce non-specific signals.

**Figure 7:**
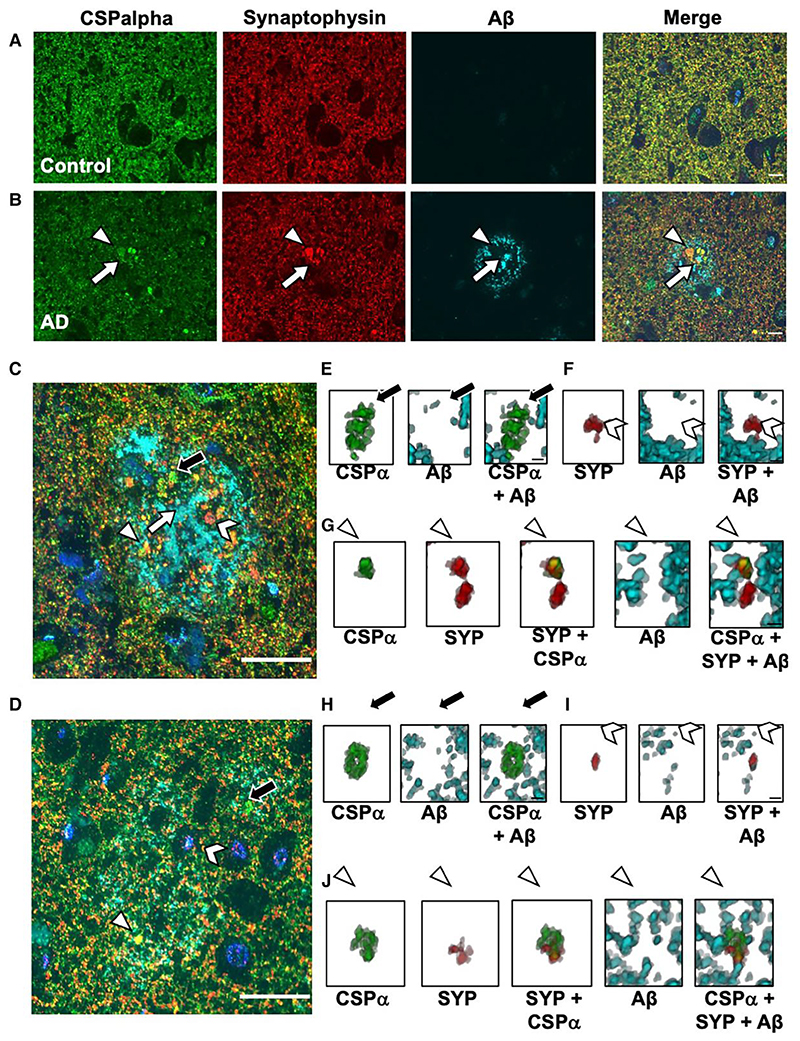
CSPalpha and synaptophysin-positive pre-synaptic dystrophies at senile and diffuse plaques. Representative array tomography images of human BA9 (**A**) control (*n* = 8) and (**B**) Braak Stage VI Alzheimer’s disease sections (*n* = 10) co-labelled with antibodies against CSPalpha (green), synaptophysin (red) and Aβ (cyan). Nuclei are stained with DAPI (blue). The arrow heads indicate CSPalpha accumulations and the complete arrows indicate the plaque core. Scale bar 10 μm. (**C**) Representative image of neuritic cored and (**D**) diffuse Aβ plaque labelled with antibodies against CSPalpha, synaptophysin and Aβ . Nuclei are stained with DAPI. Scale bar 20 μm. The complete arrow indicates the plaque core. (**E** and **H**) Representative 3D reconstructions using Image J volume viewer of a singular amorphous CSPalpha (CSP) deposit alone, Aβ alone and CSPalpha with Aβ (complete black arrows), (**F** and **I**) synaptophysin (SYP)-positive pre-synaptic dystrophy alone, Aβ alone and SYP with Aβ (chevron arrow heads) and (**G** and **J**) co-localization between CSPalpha, SYP-positive pre-synaptic dystrophy and Aβ (triangular arrow heads). (**E**–**G**) 3D volume images consist of 14 and (**H**–**J**) 15 stacks, respectively. Scale bar 1 μm.

**Figure 8:**
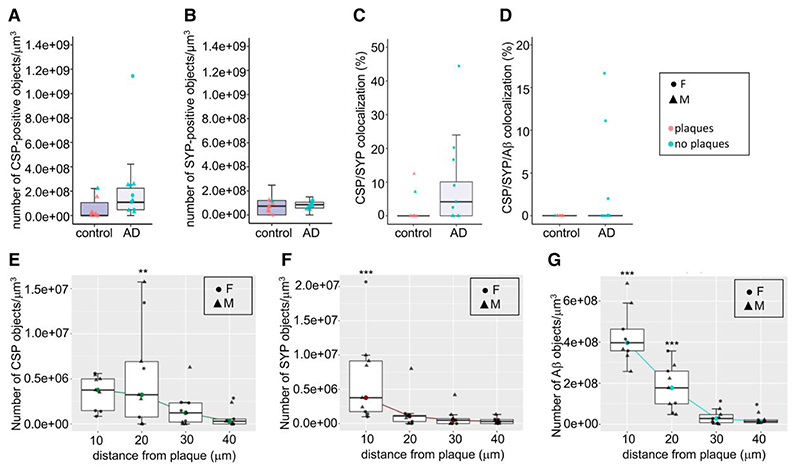
CSPalpha accumulates more distal to the plaque core than synaptophysin pre-synaptic dystrophies. Quantification of immunolabelled control (*n* = 8) and Alzheimer’s disease Braak VI (*n* = 10) BA9 brain sections, using array tomography analysis. (**A**) Alzheimer’s disease-specific differences were detected in the density of CSPalpha deposits (the number of CSP-positive objects/mm^3^) but not in the density of (**B**) pre-synaptic dystrophies [the number of synaptophysin (SYP)-positive objects/mm^3^]. (**C**) Analysis of the percentage of CSPalpha deposits that co-localized with SYP-positive pre-synaptic dystrophies (data were transformed using the Box–Cox method) and (**D**) the percentage of CSPalpha deposits co-localizing with both Aβ and SYP. Data shown are median data points per case, with boxplots showing the median for every data point and with error bars showing inter-quartile ranges. All data underwent Shapiro–Wilk’s normality testing and were analysed using a parametric linear mixed-effects model. Symbol representations: circle, female (**F**); triangle, male (**M**). Colour representations: orange, no Aβ plaques present; blue, Aβ plaques present. (**E**) Array tomography images were analysed to yield the density of synaptic accumulations at distances of 0–10 (10), 10–20 (20), 20–30 (30) and 30–40 (40) μm from the Aβ plaque core. Quantification of median CSPalpha deposit density in Braak VI brains reveals a statistically significant gain of abnormal structures approaching Aβ plaques <10 μm and at a 10–20 μm distance from the plaque core (**F**) and a statistically significant amount of synaptophysin-positive pre-synaptic dystrophies were localized <10 μm distance from the plaque core. (**G**) There was a gradual decline in Aβ levels with most statistically significant densities <10 and 10–20 μm from the plaque centre. This effect was also statistically significant for age and post-mortem delay (PMD). Data shown are median data points per case, with boxplots showing median for every data point and with error bars showing inter-quartile ranges. All data underwent Shapiro–Wilk’s normality testing, transformed using a Tukey transformation and analysed using a parametric linear mixed-effects model with *t*-tests using Satterthwaite’s method. Symbol representations: circle, female (**F**); triangle, male (**M**). **P<0.001, ***P<0.0001.

CSPalpha accumulation was again shown to be associated with both neuritic and diffuse Aβ plaques in the BA9 region of late-stage Alzheimer’s disease (Fig. 7A and B). Synaptophysin accumulations were also present surrounding the plaques (Fig. 7A and B). Pre-synaptic dystrophies in proximity to Aβ plaques can be CSPalpha-positive and/or synaptophysin negative (Fig. 7C–E, H and I). Few CSPalpha-positive and synaptophysin-positive structures co-localized with Aβ in Alzheimer’s disease and control brains (Braak VI, 3.7±1.9% and controls, 0.3 ±0.3%) (Plaque status effect; *t =* 2.053, *P =* 0.0472) (Figs 7F and J and 8D).

AT confirmed that CSPalpha accumulates in the presence of Aβ plaques in Alzheimer’s disease brain (2.6 ×10^8^±8.1 ×10^7^ objects/mm^3^) compared with the small Aβ deposits found in aged control brain (7.3 ×10^7^±4.1 ×10^7^ objects/mm^3^) (*t*=0.06, *P >* 0.05) (Fig. 8A). Plaque-associated synaptophysin accumulations, possibly linked to pre-synaptic dystrophies or degenerating synapses, were found to be more dense with increasing Aβ plaque load in Alzheimer’s disease and control brains (Braak VI, 8.3 ×10^7^±8.6 ×10^6^ objects/mm^3^ and controls, 6.9 ×10^7^±1.8 ×10^7^ objects/mm^3^) (*t*=2.84, *P*<0.01) (Fig. 8B). Of the CSPalpha structures analysed, 12.2±4.0% co-localized with synaptophysin in Alzheimer’s disease compared with control BA9 (2.6±1.7%) (Plaque status effect; *t*=5.362, *P*<0.001) (Fig. 8C).

The distances of these accumulations from the Aβ plaque centre were measured. These distances were referenced to a 30-40 μm bin, furthest away from the plaque where it would not be expected for deposits to be present (representative of a non-plaque area). CSPalpha accumulations were found highly localized at >10 μm distance (*t*=2.701, *P*<0.01) and were most abundant between 10 and 20 μm (*t*= 3.24, *P*<0.01) from the Aβ plaque core (Fig. 8E), unlike synaptophysin-positive pre-synaptic dystrophies that were found predominantly <10 μm from the plaque core (*t*= 4.152, *P*<0.001) (Fig. 8F). This confirms that CSPalpha accumulations are found distally to synaptophysin-positive pre-synaptic dystrophies relative to the plaque core.

The localization of CSPalpha and synaptophysin structures aligns with the gradual decline of Aβ oligomer concentration from the Aβ plaque with increasing distance. Significantly, most Aβ was found at <10 μm distance (*t*= 12.713, *P*<0.01) and between 10 and 20 μm (*t*= 7.60, *P*<0.01) from the plaque core (Fig. 8G), where increased amounts of both CSPalpha and synaptophysin accumulations are found. However, since only CSPalpha but not synaptophysin accumulations were found at distances >10 μm from the plaque core, this might be suggestive of different mechanisms by which abnormal synaptic structures are disrupted by Aβ, with CSPalpha-containing pre-synapses potentially more vulnerable to lower concentrations of Aβ oligomers.

## Discussion

The mechanisms underlying synaptic degeneration in Alzheimer’s disease are not well understood. Since the downregulation of CSPalpha, a molecular co-chaperone at the pre-synaptic terminal, was suggested to be a marker of early synaptic degeneration,9we performed a detailed analysis of its expression in post-mortem Alzheimer’s disease brain. We found amorphous deposits and bright focal spots of CSPalpha protein, which were localized in close proximity to Aβ plaques. These CSPalpha accumulations were detected using different antibodies against CSPalpha and with different microscopy techniques. These putative CSPalpha aggregates are evident in different regions of Alzheimer’s disease brain, including the BA9 region, hippocampus, temporal cortex and cerebellum, and they contain CSPalpha phosphorylated at Ser10. The CSPalpha accumulations are in close proximity (<20 μm) to neuritic and diffuse Aβ plaques, may be extracellular and are sometimes associated with other pre-synaptic proteins such as synaptophysin and SNAP-25.

We detected CSPalpha accumulations not only in post-mortem Alzheimer’s disease brain but also in post-mortem brain from other dementias such as FTLD and DLB. However, CSPalpha accumulations were detected only when Aβ deposits were present. In FTLD or DLB cases without Aβ plagues, no amorphous CSPalpha deposits were found. Thus, there is a strong link between CSPalpha and Aβ accumulation. This is further supported by the finding that young 5xFAD mice, which develop Aβ pathology and which do not have tau pathology,35also have CSPalpha accumulations.

In post-mortem Alzheimer’s disease brain, CSPalpha protein accumulations were most commonly found 10–20 μm from the Aβ plaque core, whereas similar synaptophysin structures were generally restricted to within 10 μm of the plaque centre. A halo of toxic Aβ oligomers directly affected pre-synaptic terminals near the edge of the Aβ plaque core,^61^ and previous array tomography studies established that the high local concentrations of soluble Aβ oligomers declined with increasing distance from plaques, returning to baseline levels within 20 μm of the plaque core.^60^ This positions CSPalpha accumulations in an area of relatively low oligomeric Aβ concentration and suggests that, compared with other pre-synaptic proteins, CSPalpha is particularly affected by synaptotoxic Aβ oligomers.

Peri-plaque pre-synaptic dystrophic neurites have previously been described in Alzheimer’s disease that lack dendritic proteins such as microtubule-associated protein 2 and β-tubulin but that accumulate various pre-synaptic proteins, including synaptophysin, SNAP-25, syntaxin, vesicular glutamate transporter 1 and vesicular inhibitory amino acid transporters.^8,50,51,53,55,56^ As a possible explanation for this, a recent proteomic analysis of APP^NLGF/NLGF^ knock-in mice determined that proteostasis is markedly disrupted in glutamatergic pre-synaptic compartments of the hippocampus and cortex from the earliest stages of plaque deposition, leading to an accumulation of several pre-synaptic proteins, including CSPalpha.^31^ APP and C-terminal fragments are enriched in plaque-associated synapses, and this is suggested to contribute to a toxic Aβ -driven feed-forward mechanism at pre-synaptic terminals.^31,62^ However, beta-secretase 1 (BACE-1)-containing pre-synaptic structures also label with synaptophysin,^53^ and only few CSPalpha accumulations co-labelled with synapto-physin or Aβ in this study, suggesting that the mechanism underlying the plaque-associated accumulation of CSPalpha is different from that of synaptophysin. This may reflect the fact that synaptophysin and CSPalpha (together with SNAP-25) are in distinct synaptic compartments that are differentially affected in an environment of high oligomeric Aβ. Thus, it is conceivable that distinct sorting mechanisms might expel synaptophysin and CSPalpha independently from pre-synaptic terminals due to oligomeric Aβ action.

Unlike synaptophysin, CSPalpha has been implicated in the export of neurodegenerative disease-associated misfolded proteins, including tau, TDP-43, and alpha-synuclein *in vitro*.*^19,29^* Indeed, CSPalpha can be co-secreted along with these misfolded proteins via an unconventional protein secretion pathway that can also include other endogenous chaperones such as DnaJB1, DnaJB11 and DnaJA1.^19,28^ This protein secretion requires CSPalpha phosphorylation at Ser10.^21^ While the mechanism of this MAPS pathway is not fully understood, tau release and its prion-like spread are exacerbated by Aβ and heightened synaptic activity.^63–65^ It is, therefore, plausible that low oligomeric Aβ concentrations are sufficient to induce release from the pre-synaptic compartments of CSPalpha that is complexed with misfolded proteins. CSPalpha self-associates into dimers, trimers and oligomers as shown *in vitro*, using purified CSPalpha.^14,66,67^ Once expelled into the extracellular spaces into an environment that promotes self-assembly of other aggregation-prone proteins, it is possible that CSPalpha self-aggregates and deposits as the amorphous structures reported here. It should be noted that CSPalpha aggregation in Alzheimer’s disease, where there is no known genetic link, would be different from CSPalpha aggregation in adult-onset neuronal ceroid lipofuscinosis (ANCL), which is caused by missense mutations in the *DNAJC5* gene.^68,69^ In ANCL, mutant CSPalpha aggregates mislocalize intracellularly, which results in CSPalpha exclusion from synapses.^69,70^ However, in Alzheimer’s disease brain, we did not find any evidence for intracellular accumulation of CSPalpha or for its exclusion from intact, synaptophysin-containing synapses.

In summary, our data show that the pre-synaptic co-chaperone CSPalpha accumulates from the earliest stages of Aβ deposition and is sensitive to low concentrations of oligomeric Aβ at ~10–20 μm from the centre of plaques. CSPalpha does not commonly appear in the same dystrophic compartments as synaptophysin, rather we suggest that CSPalpha accumulations result from its co-secretion with misfolded proteins in response to Aβ and extracellular self-aggregation. These data indicate that low oligomeric Aβ concentrations are sufficient to affect pre-synaptic terminals, which is consistent with publications showing that synaptic degeneration in Alzheimer’s disease originates at the presynapse. Furthermore, our results show that CSPalpha is not only a more sensitive marker for synaptic loss and/or early pre-synaptic dysfunction compared with traditional synaptic markers in dementia, but that CSPalpha should be used as a pathological marker for early synaptic disruption caused by Aβ. A future study of CSPalpha levels in human cerebrospinal fluid19may also provide a sensitive peripheral biomarker of Alzheimer’s disease stage and/or the efficacy of new Alzheimer’s disease therapeutics.

## Supplementary Material

Supplementary figures

Supplementary tables

## Data Availability

Raw data are available from the authors upon reasonable request.

## Abbreviations

Aβ: beta-amyloid
ANCL: adult-onset neuronal ceroid lipofuscinosis
APP: amyloid precursor protein
BA9: Brodmann area 9
BACE1: beta-secretase 1
BCA: bicinchoninic acid assay
CSPalpha: cysteine string protein alpha
DAPI: 4′,6-diamidino-2-phenylindole
DLB: dementia with Lewy bodies
ER: endoplasmic reticulum
FTLD: fronto-temporal lobar dementia
GFAP: glial fibrillary acidic protein
HEPES: 4-(2-hydroxyethyl)-1-piperazineethanesulfonic acid
HSc70: heat shock cognate 71 kDa protein
Iba1: ionized calcium binding adaptor molecule 1
iSIM: instant Structured Illumination Microscopy
MAPS: misfolding-associated protein secretion
NGS: normal goat serum
PBS: phosphate-buffered saline
PMD: post-mortem delay
PS1: presenilin 1
SDS: sodium dodecyl sulphate
Ser: serine
SNAP-25: synaptosome-associated protein 25 kDa
TBS: Tris-buffered saline
