# Supplementary material for: Cysteine string protein alpha accumulates with early pre-synaptic dysfunction in Alzheimer’s disease
Source: Brain Commun. Author manuscript; Available in PMC 2022 Aug 9. (PMC9345313; doi:10.1093/braincomms/fcac192)

Supplementary Figure 1

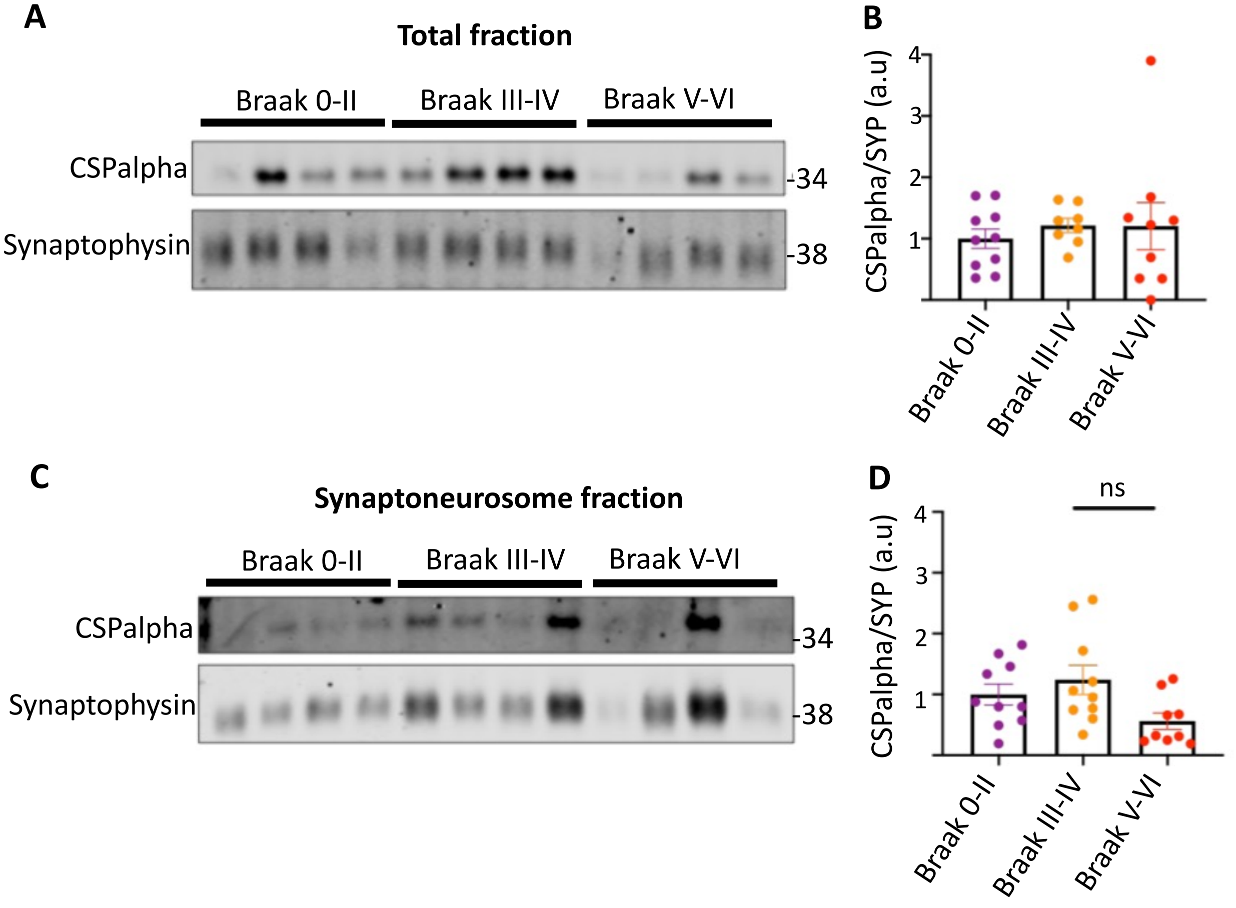

Supplementary Figure 1. CSPalpha levels in total and synaptic fractions from BA9.

(A) Representative western blots of total homogenates from Braak stages 0-II, III-IV and V-VI post-mortem BA9 cortex. Membranes were probed with antibodies against CSPalpha and synaptophysin. **(B)** Bar charts show quantification of CSPalpha amounts relative to synaptophysin amounts in the same sample. Following D'Agostino and Pearson normality testing, data were analysed using nonparametric Kruskal–Wallis tests. (**C**) Representative western blots of the synaptoneurosome fraction probed with antibodies against CSPalpha and synaptophysin. (**D**) Bar charts show quantification of CSPalpha amounts in BA9 synaptoneurosomes when controlled for synaptic content by normalising to synaptophysin levels in the same sample. Following D'Agostino and Pearson normality testing, data were analysed using a one-way ANOVA. Data shown are mean +/- SEM expressed as fold average control. NS = Not statistically significant. [n = 10 cases per group].

Supplementary Figure 2

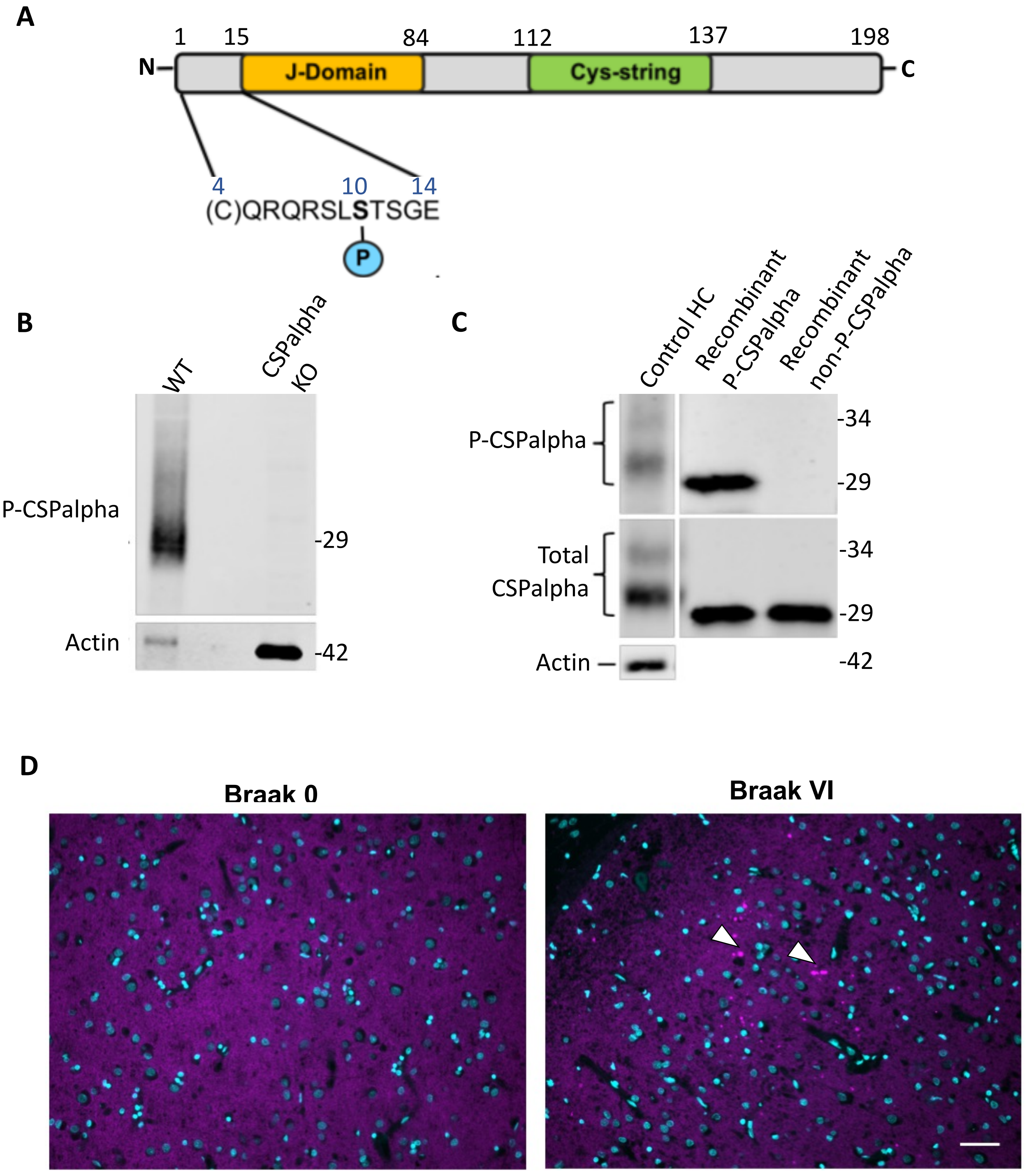

Supplementary Figure 2. CSPalpha that deposits in AD brain is phosphorylated at Ser10.
(**A**) Schematic representation of CSPalpha indicating the J-domain, cysteine string and the location of the PKA Ser10 phosphorylation site including the peptide sequence.

Phospho-CSPalpha (4–14) was used to raise the antibody, with an additional N-terminal cysteine to facilitate coupling. Representative western blots showing **(B)** detection of CSPalpha pSer10 (P-CSPalpha) in WT, but not CSPalpha KO forebrain confirming antibody specificity to CSPalpha. Beta-actin was used as a loading control. **(C)** Representative western blots showing detection of only phosphorylated, but not non-phosphorylated recombinant CSPalpha peptides by the phospho-CSPalpha antibody. CSPalpha pSer10 was also detected in human hippocampus. Due to the lack of posttranslational modifications, recombinant protein appears to be of lower molecular weight than CSPalpha from tissue. **(D)** Representative images of post-mortem human BA9 from control (Braak stage 0) and AD (Braak stage VI) brain immunolabelled with antiserum against CSPalpha pSer10 (magenta). Nuclei are stained with DAPI (cyan). White arrow heads indicate CSPalpha accumulations immunolabelled with an antibody specific to CSPalpha phosphorylated at Ser10. Scale bar 50 µm. [representative images from n = 5 cases per group].

Supplementary Figure 3

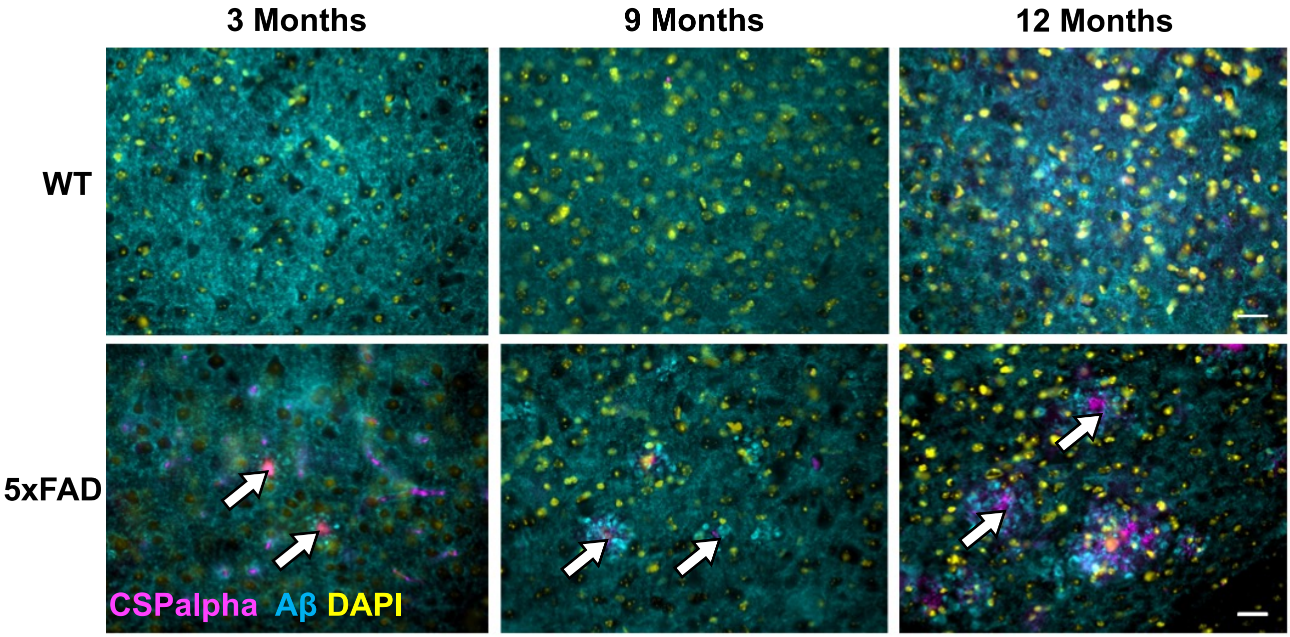

Supplementary Figure 3. CSPalpha accumulations in 5xFAD mice. Representative images of sections taken from the cortex of WT and 5xFAD mice at 3-, 9- and 12-months of age. Images show the progressive appearance of 6E10-labelled Aβ deposits (cyan) alongside the accumulation and of CSPalpha deposits (magenta). DAPI stain (yellow) was used to identify nuclei. Scale bar 25 μm. [representative images from n = 4 WT and n=3 5xFAD mice, minimum of 3 sections per animal].

Supplementary Figure 4

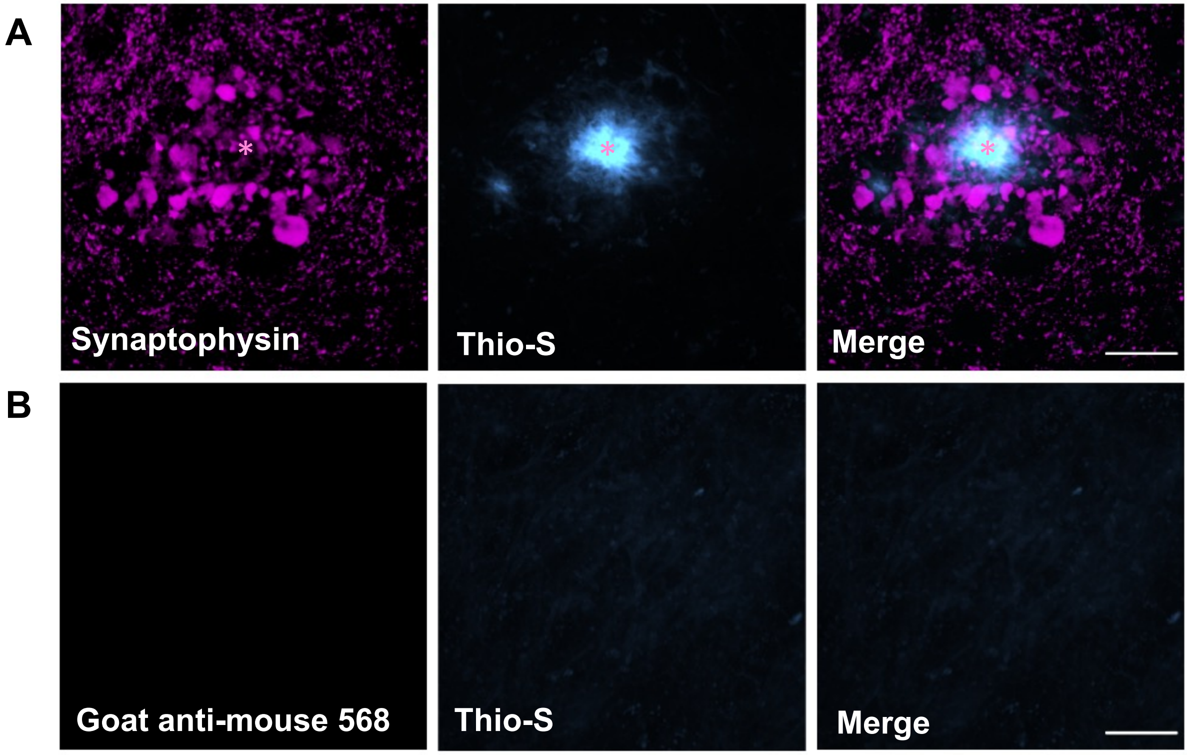

Supplementary Figure 4. Synaptophysin accumulations are localised to fibrillar Aβ deposits in 5xFAD mice. (A) Representative images of hippocampal sections from 5xFAD mice, co-labelled using an antibody against synaptophysin (magenta) which accumulates in the periphery of ThioS labelled Aβ plaques (cyan). **(B)** Representative images of negative control of a WT mouse hippocampal section probed only with secondary antibodies for Alexa-fluor 568. Asterisks indicate plaque core. Scale bar 20 μm. [representative images from n = 4 WT and n=3 5xFAD mice, minimum of 3 sections per animal].

Supplementary Figure 5: Uncropped blots

A: Used in Figure 2B

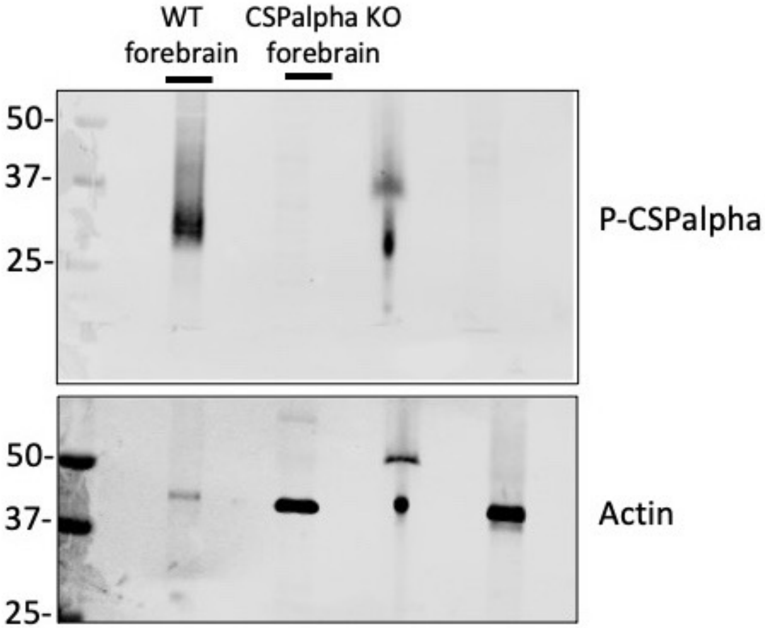

B: Used in Figure 2C

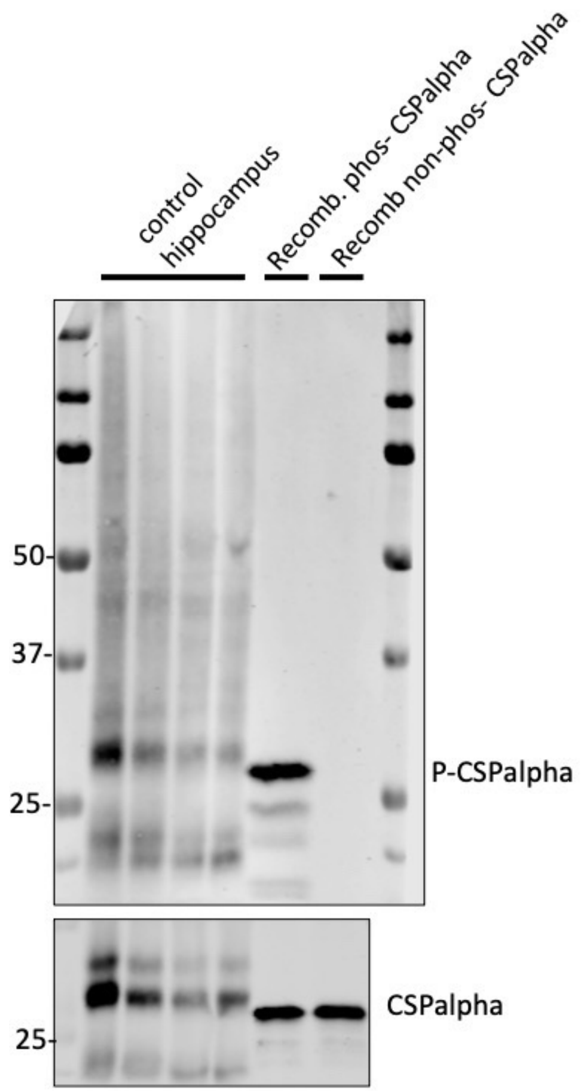

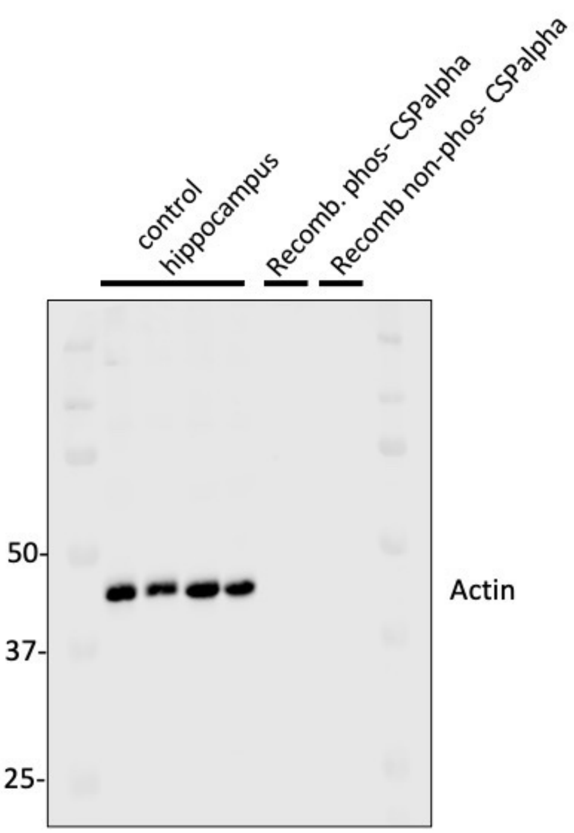

Supplement: Supplementary figures [file EMS151722-supplement-Supplementary_figures.pdf]
