# Supplementary material for: Cysteine string protein alpha accumulates with early pre-synaptic dysfunction in Alzheimer’s disease
Source: Brain Commun. Author manuscript; Available in PMC 2022 Aug 9. (PMC9345313; doi:10.1093/braincomms/fcac192)
Supplement: Supplementary tables [file EMS151722-supplement-Supplementary_tables.pdf]

Supplementary Table 1

Characteristics of human tissue brains used in this study.

Table shows details of primary pathological diagnosis, sex, age, PMD, Braak/BNE stage and region for tissue used.

(CAA: cerebral amyloid angiopathy, AD: Alzheimer's Disease, SVD: small vessel disease, WM: White matter)

Case Number	MRC BBN	Pathological Diagnosis	Brain Region	Braak/BNE Stage	PMD (hours)	Sex (M/F)	Age at Death (years)
**1**	BBN_15777	No significant abnormalities	BA9	0	21.5	F	87
**2**	BBN_15616	No significant abnormalities	BA9	0	6	M	86
**3**	BBN_1571	No significant abnormalities	BA9	0	50	M	59
**4**	BBN_15790	No significant abnormalities	BA9	0	40	M	40
**5**	BBN_15734	Mild age-related changes	BA9	0	9.5	M	78
**6**	BBN_16368	Argyrophilic grains low to moderate density and early Alzheimer-type neurofibrillary lesions	BA9	II	13	F	82
**7**	BBN 001.35823	Mild CAA only		-	72	F	40

			BA9				
**8**	BBN 001.34215	No significant abnormalities	BA9	0	49	M	50
**9**	BBN 001.32577	Mild SVD,<br>WM microinfarcts,<br>Mild CAA	BA9	II	74	M	81
**10**	BBN 001.30972	No significant abnormalities	BA9	0	99	M	34
**11**	BBN 001.30140	Mild WM pathology,<br>Mild non-amyloid SVD	BA9	0	122	M	50
**12**	BBN 001.33614	Mild WM pathology	BA9	0	76	M	46
**13**	BBN 001.35529	No significant abnormalities	BA9	0	96	M	58
**14**	BBN 001.30841	Mild non-amyloid SVD	BA9	-	103	M	40
**15**	BBN_14408	Mild age-related changes (control brain) - mild focal CAA	BA9	0	45	M	90
**16**	BBN_16236	Control case with Hypoxic-type changes and mild CAA	BA9	I-II	41	F	89

**17**	BBN_4581	Ageing changes	BA9, HC, CB	II	24	M	82
**18**	BBN_22594	Control brain	BA9, HC, CB	0	21	F	77
**19**	BBN_18816	Old infarcts in the right cerebral hemisphere	HC, CB	0	53	M	84
**20**	BBN_22991	Early ageing changes	BA9	I	27	F	73
**21**	BBN_9924	Alzheimer-type changes with CAA	BA9	III	12.5	M	81
**22**	BBN002.28871	AD with moderate CAA; moderate to severe cerebrovascular changes; limbic TDP-43 pathology (Joseph stage II)	BA9	IV	47	F	95
**23**	BBN002.28694	AD with mild CAA	BA9	IV	55.5	F	86
**24**	BBN_24549	AD with mild CAA	BA9	IV	53	M	98
**25**	BBN_15210	AD with limbic TDP-43 pathology	BA9	IV	28	M	82
**26**	BBN_9881	AD with mild CAA	BA9	V	12.5	F	80

**27**	BBN_9775	AD with moderate CAA	BA9	V	26	M	86
**28**	BBN_9796	AD	BA9	V	<24	F	84
**29**	BBN_9829	AD	BA9	V	12	F	97
**30**	BBN_9855	AD with severe CAA	BA9	VI	41	M	80
**31**	BBN_4197	AD	BA9, HC, CB	VI	26.5	F	83
**32**	BBN_9928	AD with severe CAA	BA9, HC, CB	VI	5.5	M	72
**33**	BBN002.30132	AD	BA9	VI	48	F	95
**34**	BBN002.30130	AD; moderate cerebrovascular pathology; limbic and early cortical TDP-43 pathology	BA9	VI	27	M	85
**35**	BBN002.28697	AD with moderate CAA and dementia with Lewy bodies (limbic stage) and limbic TDP-43 pathology	BA9, HC, CB	VI	38.5	F	89
**36**	BBN 001.33636	AD, severe WM pathology, Severe non-amyloid SVD, Lewy Body Disease (brainstem)	BA9	VI	43	M	93
**37**		AD, severe WM pathology,	BA9	VI	80	F	85

	BBN 001.32929	Severe non amyloid SVD<br>Severe arteriolar CAA					
**38**	BBN 001.30973	AD,<br>Severe arteriolar CAA<br>moderate WM pathology,<br>Moderate non-amyloid SVD	BA9	VI	96	F	89
**39**	BBN 001.31499	AD,<br>moderate WM pathology,<br>Mild non-amyloid SVD<br>Mild CAA<br>Lewy Body Disease<br>(brainstem)	BA9	VI	78	M	85
**40**	BBN<br>001.35182	AD,<br>mild WM pathology,<br>Mild non-amyloid SVD<br>Severe arteriolar CAA	BA9	VI	49	M	66
**41**	BBN 001.31495	AD,<br>mild WM pathology, Mild non-amyloid SVD<br>Mild arteriolar CAA	BA9	VI	38	M	81
**42**	BBN 001.35564	AD,<br>Moderate arteriolar CAA<br>mild WM pathology,<br>Mild non-amyloid SVD<br>Lewy Body Disease<br>(brainstem)	BA9	VI	52	F	90

**43**	BBN 001.33698	AD,<br>Severe arteriolar CAA<br>moderate WM pathology,<br>Severe non-amyloid SVD,<br>Lewy Body Disease<br>(brainstem)	BA9	VI	76	F	90
**44**	BBN 001.29695	AD,<br>severe WM pathology,<br>Severe non-amyloid SVD,<br>Moderate arteriolar CAA,<br>Lewy Body disease (limbic)	BA9	VI	72	M	86
**45**	BBN 001.30883	AD,<br>Moderate arteriolar CAA,<br>severe WM pathology,<br>Moderate non-amyloid SVD	BA9	VI	69	F	61
**46**	BBN 001.28410	AD<br>mild WM pathology,<br>Moderate non-amyloid SVD,<br>Mild arteriolar CAA	BA17	VI	109	F	62
**47**	BBN_24681	FTLD with motor neurone disease (FTLD-MND) subtype B	Temporal Cortex	V	67	F	71
**48**	BBN_10245	FTLD-TDP (type A) with mild to moderate vascular pathology	Temporal Cortex	III	31	M	87

**49**	BBN002.26654	FTLD-TDP (type A), Lewy Body Disease (limbic).	Temporal Cortex	-	48.5	F	91
**50**	BBN002.28594	FTLD-TDP (type A)	Temporal Cortex	-	66	M	72
**51**	BBN_24396	FTLD-TDP (type A), AD	Temporal Cortex	IV	34	F	71
**52**	BBN_16241	Lewy Body Disease (limbic)	Temporal Cortex	III	48	F	79
**53**	BBN_16400	Lewy Body Disease (diffuse neocortical)	Temporal Cortex	-	35	M	74
**54**	BBN_16353	Lewy Body Disease (diffuse neocortical)	Temporal Cortex	-	18	M	74
**55**	BBN_24626	AD;<br>Lewy Body Disease (diffuse neocortical)	Temporal Cortex	IV	16	F	87
**56**	BBN_13797	Lewy Body Disease (early neocortical)<br>mild AD	Temporal Cortex	II	40	M	66

Supplementary Table 2

Primary antibodies used for immunofluorescence and array tomography.

Antibody	Epitope and Specificity	Species	Dilution	IF / AT	Source
6E10	Anti-β-amyloid Amino acids 1-16 of human Aβ sequence	Mouse	1/500	IF / AT	803001, Biolegend, UK
AT8	Tau phosphorylated at Ser202/Thr205	Mouse	1/250	IF	MN1020, Thermoscientific, UK
CSPalpha	Raised against recombinant rat CSP with deletion of cysteine string	Rabbit	1/250	IF / AT	AB1576, Merck Millipore Ltd., UK
GFAP	Glial fibrillary acidic protein (GFAP)	Mouse	1/250	IF	VP-G805, Vector Laboratories
Hsc70	Amino acids 583-601 at C-terminus of human Hsc70	Mouse	1/1000	IF	sc-7298, Santa Cruz, UK
IBA-1	Peptide corresponding to 20 amino acids from N-terminal region of human Iba1/AIF1	Mouse	1/200	IF	MABN92, Merck Millipore Ltd., UK
Phosphorylated CSPalpha	CSPalpha Phosphorylated at Ser10	Rabbit	1/50	IF	Kind gift from Prof Alan Morgan (University of Liverpool, UK)
SMI312	Homogenised hypothalamic recovered from Fischer 344 rats	Mouse	1/500	IF	Kind gift from Prof Chris Miller (King's College London, UK)
SNAP-25	Raised against post-mortem human brain	Mouse	1/250	IF	sc-20038, Santa Cruz, UK
Synaptophysin	Amino acids 221-313 of synaptophysin of human origin	Mouse	1/250	IF	sc-17750, Santa Cruz, UK

Synaptophysin	*E. coli*-derived recombinant human synaptophysin	Goat	1/50	AT	AF5555, R&D Systems, UK

Supplemantary Table 3

Secondary antibodies used for immunofluorescence and array tomography.

Secondary Antibody	Wavelength	Dilution	IF / AT	Source
Alexa Fluor Goat anti Rabbit	488	1/250	IF	A11034, Thermofisher Scientific, UK
Alexa Fluor Goat anti Mouse	568	1/250	IF	A11004, Thermofisher Scientific, UK
Alexa Fluor Donkey anti Rabbit	488	1/50	AT	A21207, Thermofisher Scientific, UK
Alexa Fluor Donkey anti Mouse	568	1/50	AT	A21202, Thermofisher Scientific, UK
Alexa Fluor Donkey anti Goat	647	1/50	AT	A21447, Thermofisher Scientific, UK
